# Kidney Function and Mortality in Mexico: Prospective Study of 130,000 Adults

**DOI:** 10.1016/j.xkme.2026.101398

**Published:** 2026-05-12

**Authors:** Diego Aguilar-Ramirez, Roberto Tapia-Conyer, William G. Herrington, Raúl Ramirez-Reyes, Adrián Garcilazo-Ávila, Carlos González-Carballo, Rogelio Santacruz-Benitez, Omar Yaxmehen Bello-Chavolla, Fiona Bragg, Louisa Gnatiuc Friedrichs, Michael Hill, Lisa Holland, Jason Torres, Eirini Trichia, Michael Turner, Natalie Staplin, Rachel Wade, Doreen Zhu, Rory Collins, Richard Haynes, Richard Peto, Jaime Berumen, Jesus Alegre-Díaz, Pablo Kuri-Morales, Jonathan R. Emberson

**Affiliations:** 1 Clinical Trial Service Unit & Epidemiological Studies Unit, Nuffield Department of Population Health, University of Oxford, Oxford, UK; 2 Faculty of Medicine, National Autonomous University of Mexico, Mexico City, Mexico; 3 Experimental Research Unit from the Faculty of Medicine, National Autonomous University of Mexico, Mexico City, Mexico; 4 Research Division, Instituto Nacional de Geriatría, Mexico City, Mexico; 5 Health Data Research UK Oxford, University of Oxford, Oxford, UK; 6 Tecnológico de Monterrey, Proyecto OriGen, Monterrey, Mexico

**Keywords:** TK, kidney function, cause-specific mortality, prospective study, Mexico

## Abstract

**Rationale & Objective:**

Reduced kidney function is strongly associated with higher mortality, but most evidence is from high-income populations. This study evaluated the relevance of kidney function to cause-specific mortality in Mexico, a country where diabetes is common and chronic kidney disease (CKD) is a major cause of morbidity and mortality.

**Study Design:**

Prospective study of Mexican adults aged ≥35 years at recruitment (1998-2004) who were followed until October 2022.

**Setting & Participants:**

Participants recruited into the Mexico City Prospective Study. Analyses focused on 126,245 participants aged 35-74 years at recruitment without prior disease (except diabetes or CKD).

**Exposures:**

Estimated glomerular filtration rate (eGFR).

**Outcomes:**

Cause-specific mortality.

**Analytical Approach:**

Cox regression was used to relate eGFR to cause-specific mortality. Analyses were adjusted for sociodemographic and lifestyle factors, anthropometry, and diabetes.

**Results:**

Among 40,996 men and 85,249 women aged 35-74 years, median eGFR was 102 (IQR, 91-110) mL/min/1.73 m^2^, mean body mass index was 29.1 (SD 4.9) kg/m^2^, 1% had self-reported CKD, 13% had previously diagnosed diabetes, and 12,590 died at ages 35-74 years over a median follow-up of 20.4 years. For those with eGFR <105 mL/min/1.73 m^2^, each 15-mL/min/1.73 m^2^ lower eGFR was associated with 32% higher all-cause mortality (RR, 1.32; 95% CI, 1.30-1.35). The strongest associations were for kidney (RR, 1.75; 95% CI, 1.69-1.80), infective (RR, 1.34; 95% CI, 1.24-1.44), and vascular deaths (RR, 1.28; 95% CI, 1.24-1.33). Compared with participants with eGFR 90-104 mL/min/1.73 m^2^, those with eGFR <30 mL/min/1.73 m^2^ had almost 7 times the all-cause mortality rate (RR, 6.5; 95% CI, 5.7-7.3). For participants with eGFR >105 mL/min/1.73 m^2^, higher eGFR was associated with higher mortality. The absolute excess mortality risk associated with reduced eGFR was particularly high for those with diabetes.

**Limitations:**

Data on urinary albumin and nonfatal disease outcomes were unavailable.

**Conclusions:**

In Mexico, decreased kidney function is strongly associated with premature mortality, mainly from vascular, kidney, and infective causes. Prevention and management of CKD, particularly in individuals with diabetes, should be central to disease-prevention policies.

## Plain-Language Summary

Evidence of the impact of decreased kidney function on the risk of death is lacking in Mexico, a country where diabetes and kidney disease are major causes of death. Between 1998 and 2004, the Mexico City Prospective Study recruited 150,000 adults aged ≥35 years and tracked their causes of death for over 20 years. This study found that decreased kidney function was linked with a higher risk of death, mainly because of heart disease, kidney disease, and infections. Individuals with substantially decreased kidney function were about 7 times more likely to die than those with apparently healthy kidney function. Having both diabetes and decreased kidney function resulted in an extremely high risk of death. Increasing the use of kidney and heart-protective drugs in people with diabetes, elevated blood pressure, or chronic kidney disease and expanding access to dialysis and kidney transplantation will likely reduce premature mortality in Mexico.

## Graphical abstract

**Figure:**
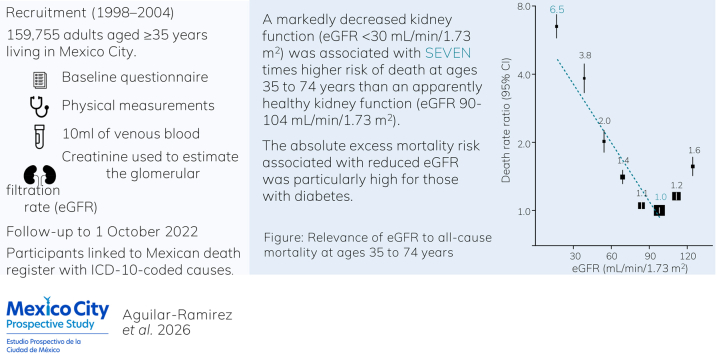

Chronic kidney disease (CKD) is defined by a glomerular filtration rate (GFR) <60 mL/min/1.73 m^2^ or the presence of markers of kidney damage, such as albuminuria, that persists for ≥3 months with implications for health.^1^ According to the Global Burden of Disease, in 2017 about 700 million adults had CKD and the estimated global prevalence was 9.1%.^2^ In Mexico, a middle-income country in Latin America, the estimated prevalence of CKD is higher (12.1%) with about 15 million adults having CKD.^2^ Recently, the 78th World Health Organization assembly acknowledged kidney disease as a priority noncommunicable disease,^3^^,^^4^ alongside cardiovascular diseases, diabetes, cancer, respiratory diseases, and mental illness, recognizing its rising burden and disproportionate impact on people living in low- and middle-income countries.

Meta-analyses of prospective studies, mostly in the United States, Europe, Asia, and Australia, have shown that CKD is associated with increased risk of progression to kidney failure and of all-cause and cardiovascular mortality.^5^^,^^6^ However, no large-scale prospective study has evaluated the association of CKD with mortality in Mexico. In Mexico, where by the early 2000s around one fifth of adults aged 60 years had been diagnosed with diabetes^7^ and obesity affected around half of women and one third of men,^8^ diabetes has a greater impact on mortality than in many other populations,^9^ with the largest effects seen for renal mortality. This unduly large excess risk is mostly explained by poor glycemic control^10^ and suboptimal management of comorbid conditions and other risk factors.^11^ Reports from a CKD screening program in Mexico showed that early signs of CKD are present in up to a third of those with hypertension or diabetes, the most common causes of CKD in this population, but CKD is often underdiagnosed and undertreated,^12^ likely leading to unnecessarily high risks of adverse outcomes and death. Importantly, mortality rates because of kidney disease have more than doubled in Mexico over the last 30 years and are among the highest worldwide.^13^

Given the lack of population-specific large-scale evidence of the association between kidney function and mortality in Mexico, there is uncertainty whether its impact is the same as in high-income countries where diabetes and CKD are generally better managed. Direct evidence from Mexico would both facilitate reliable estimation of the burden of CKD in this population and provide valuable insights on the consequences of reduced kidney function for many other populations where diabetes and obesity may be increasing but have not yet reached the levels seen in Mexico.

In this study, we used data from approximately 130,000 men and women from the Mexico City Prospective Study who have been followed for 2 decades to assess the association between estimated GFR (eGFR) and all-cause and cause-specific mortality, and in particular, how these associations varied depending on age, sex, and diabetes.

## Methods

The Mexico City Prospective Study design and methods have been described previously.^14^ A detailed description, including data collection, assessment of kidney function, and mortality follow-up procedures and outcomes for the current report is provided in the Supplementary Methods (Item S1). The statistical methods are described below.

Analyses excluded participants aged 85 years or older and those with missing data on creatinine, missing covariate data (see below), or uncertain cause of death (defined as a ≥1 year discrepancy in a participant’s date of birth as recorded at the baseline survey compared with the matched death certificate). Those with a self-reported prior diagnosis at recruitment of coronary heart disease, cerebrovascular disease, cancer, cirrhosis, or emphysema were further excluded to limit the risk of reverse causation bias, whereby prior diseases may alter creatinine level (which to some extent depends on muscle mass) before enrollment. Participants were categorized into 8 baseline-defined eGFR groups (broadly reflecting the thresholds used for CKD clinical staging) using the Chronic Kidney Disease Epidemiology Collaboration (CKD-EPI) 2009 creatinine equation: <30 mL/min/1.73 m^2^, ≥30 to <45 mL/min/1.73 m^2^, ≥45 to <60 mL/min/1.73 m^2^, ≥60 to <75 mL/min/1.73 m^2^, ≥75 to <90 mL/min/1.73 m^2^, ≥90 to <105 mL/min/1.73 m^2^ (reference group), ≥105 to <120 mL/min/1.73 m^2^, and ≥120 mL/min/1.73 m^2^. Some analyses classified participants into fewer eGFR groups to preserve statistical power for between-group comparisons. Movement across these categories between the baseline and resurvey assessments was also assessed.

Cox proportional hazards regression models, with time since entry into the study as the underlying timescale, were used to assess the relevance of eGFR for all-cause and cause-specific mortality. Participants who did not die from the cause of interest were censored at the earliest of death from any other cause, the end of the age-at-risk period of interest, or October 1, 2022 (see Item S1: Supplementary Methods for details on follow-up for mortality). The log hazard ratio from a Cox model provides a useful summary statistic for the average log mortality rate ratio (RR) across the different time periods of follow-up, even if the true RRs across different time periods vary (ie, if there is nonproportionality of hazards). These mortality RRs were stratified by age-at-risk (5-year groups) and adjusted for sex, socioeconomic status, lifestyle factors (smoking, alcohol consumption, and physical activity), adiposity, and diabetes (including the extent of glycemic control) (see Item S1: Supplementary Methods for details on covariate categories), which we considered potential confounders. To explore whether the average mortality RR varied across the different time periods, RRs associated with eGFR were estimated separately for deaths occurring in the first 5 years of follow-up and deaths occurring after 5 years. Subgroup analyses were done by age-at-risk, sex, and diabetes (no diabetes vs previously diagnosed or undiagnosed). For plotting, group-specific confidence intervals (CIs) around the RRs (including for the reference group with RR of 1.0) were estimated using the variance of the log risk in each group.^15^ The main analyses examined ‘premature’ mortality (which we defined as death before age 75 years), but the relevance of eGFR for mortality at ages 75-84 years was also examined.

Subsequent analyses, with the same adjustment for covariates as described above, included eGFR as a continuous variable (per 15-mL/min/1.73 m^2^ lower or higher eGFR). Because of clear evidence for nonlinear risk relationships, these RRs were estimated separately: (1) for participants with an eGFR <105 versus ≥105 mL/min/1.73 m^2^; and (as sensitivity analyses) (2) for participants with an eGFR <90 versus ≥90 mL/min/1.73 m^2^. All-cause and cause-specific mortality RRs associated with eGFR also were estimated in models further adjusted for potential mediators of the associations between eGFR and mortality and for potential markers of frailty. These included baseline levels of blood pressure (systolic and diastolic), standard lipids (low-density lipoprotein cholesterol, high-density lipoprotein cholesterol, total triglycerides, apolipoprotein B, and apolipoprotein A1), albumin, and glycoprotein A (a biomarker of chronic inflammation^16^, ^17^, ^18^), with each of these divided into 4 equally sized groups according to the sex-specific distributions. Sensitivity analyses included estimation of all-cause mortality RRs including participants with self-reported chronic diseases and analyses that used the CKD-EPI 2021 equation to calculate eGFR rather than the CKD-EPI 2009 equation.

## Results

### Included Participants

Of the 159,755 participants recruited, 20,936 (13%) were excluded from all analyses. These comprised 238 (<0.1%) participants who were recruited twice (data from the first visit at which a blood sample was collected were used), a further 2,460 (2%) who were aged ≥85 years at recruitment, a further 13,045 (8%) with missing baseline creatinine values, a further 2,880 (2%) with missing or implausible covariate data, and a further 2,313 (1%) with uncertain mortality linkage. Of the remaining 138,819 participants, 5,471 (4%) had self-reported chronic diseases at recruitment (except diabetes or CKD) and were further excluded from the main analyses, leaving 133,348 participants, 126,245 (95%) aged 35-74 years and 7,103 (5%) aged 75-84 years.

### Baseline Characteristics by eGFR Levels

Among the 126,245 participants aged 35-74 years at recruitment, 85,249 (68%) were women, and the mean age was 50 (standard deviation 11) years (Table 1). Overall, 999 (1%) reported having previously diagnosed chronic kidney insufficiency (CKI; there was no internationally standardised definition of CKD until 2002), of which 219 (22%) had an eGFR <60 mL/min/1.73 m^2^. eGFR was <30 mL/min/1.73 m^2^ for 519 (0.4%), ≥30 to <60 mL/min/1.73 m^2^ for 2,031 (2%), ≥60 to <90 mL/min/1.73 m^2^ for 25,979 (21%), ≥90 to <105 mL/min/1.73 m^2^ for 46,368 (37%), ≥105 to <120 mL/min/1.73 m^2^ for 45,625 (36%), and ≥120 mL/min/1.73 m^2^ for 5,723 (5%) participants.

**Table 1:** Baseline Characteristics of 126,245 Participants Without Prior Chronic Disease (except Diabetes and CKD) and Aged 35-74 at Recruitment, According to eGFR Levels

	eGFR, mL/min/1.73 m^2^						Overall
	<30 (n = 519)	≥30 to <60 (n = 2,031)	≥60 to <90 (n = 25,979)	≥90 to <105 (n = 46,368)	≥105 to <120 (n = 45,625)	≥120 (n = 5,723)	(N = 126,245)
Age, y	61 (10)	63 (9)	58 (11)	54 (9)	43 (6)	39 (4)	50 (11)
Men	194 (37%)	620 (31%)	8,745 (34%)	16,338 (35%)	13,426 (29%)	1,673 (29%)	40,996 (32%)
Resident of Coyoacán	165 (32%)	722 (36%)	9,695 (37%)	19,231 (41%)	19,144 (42%)	2,207 (39%)	51,164 (41%)
University/high school educated	33 (6%)	159 (8%)	3,581 (14%)	6,372 (14%)	9,103 (20%)	1,037 (18%)	20,285 (16%)
Current smoker	112 (22%)	447 (22%)	7,507 (29%)	14,593 (31%)	16,610 (36%)	2,071 (36%)	41,340 (33%)
Current drinker	188 (36%)	1,091 (54%)	16,961 (65%)	31,605 (68%)	32,169 (71%)	3,907 (68%)	85,921 (68%)
Any regular leisure-time physical activity	61 (12%)	433 (21%)	6,618 (25%)	10,758 (23%)	9,341 (20%)	941 (16%)	28,152 (22%)
Creatinine^a^, μmol/L	297 (211-508)	108 (95-125)	76 (69-86)	64 (57-71)	57 (51-63)	45 (41-50)	63 (55-73)
eGFR^b^, mL/min/1.73 m^2^	16 (8-23)	53 (45-57)	82 (75-87)	98 (94-102)	111 (108-114)	123 (121-126)	102 (91-110)
Self-reported CKI	153 (29%)	66 (3%)	226 (1%)	259 (1%)	271 (1%)	24 (<0.5%)	999 (1%)
Physical measurements							
BMI, kg/m^2^	27.6 (4.9)	29.0 (4.9)	29.3 (4.7)	29.3 (4.9)	29.0 (5.1)	28.4 (5.3)	29.1 (4.9)
Waist circumference, cm	96 (12)	97 (11)	96 (11)	95 (11)	92 (12)	91 (12)	94 (11)
Hip circumference, cm	102 (11)	105 (11)	105 (10)	105 (10)	104 (11)	103 (11)	105 (11)
SBP, mm Hg	149 (24)	138 (20)	130 (17)	128 (16)	123 (14)	121 (13)	127 (16)
DBP, mm Hg	91 (13)	86 (11)	84 (10)	84 (10)	81 (10)	80 (9)	83 (10)
Diabetes status							
No diabetes	153 (29%)	1,076 (53%)	20,608 (79%)	37,284 (80%)	39,979 (88%)	5,006 (87%)	104,106 (82%)
Undiagnosed diabetes^c^	5 (1%)	90 (4%)	1,250 (5%)	2,562 (6%)	1,895 (4%)	286 (5%)	6,088 (5%)
Previously diagnosed diabetes	361 (70%)	865 (43%)	4,121 (16%)	6,522 (14%)	3,751 (8%)	431 (8%)	16,051 (13%)
HbA_1c_ <9%	304 (59%)	515 (25%)	2,336 (9%)	3,290 (7%)	1,514 (3%)	138 (2%)	8,097 (6%)
HbA_1c_ 9 to <11%	30 (6%)	199 (10%)	965 (4%)	1,698 (4%)	1,046 (2%)	126 (2%)	4,064 (3%)
HbA_1c_ ≥11%	27 (5%)	151 (7%)	820 (3%)	1,534 (3%)	1,191 (3%)	167 (3%)	3,890 (3%)
Taking glucose-lowering medication	274 (53%)	743 (37%)	3,371 (13%)	5,199 (11%)	2,904 (6%)	319 (6%)	12,810 (10%)
HbA_1c_, %	6.7 (2.0)	7.0 (2.2)	6.2 (1.7)	6.2 (1.7)	5.9 (1.6)	5.9 (1.7)	6.1 (1.7)
Other medication use							
Any antihypertensive	336 (65%)	865 (43%)	5,751 (22%)	7,086 (15%)	2,803 (6%)	233 (4%)	17,074 (14%)
Renin angiotensin system inhibitor	237 (46%)	655 (32%)	4,260 (16%)	5,294 (11%)	2,134 (5%)	162 (3%)	12,742 (10%)
Any antithrombotic	14 (3%)	77 (4%)	959 (4%)	1,158 (2%)	697 (2%)	58 (1%)	2,963 (2%)
Any lipid-lowering	4 (1%)	34 (2%)	229 (1%)	265 (1%)	112 (<0.5%)	5 (<0.5%)	649 (1%)
Lipids and other biomarkers							
LDL cholesterol, mmol/L	2.5 (1.1)	2.6 (1.0)	2.6 (0.8)	2.5 (0.8)	2.4 (0.7)	2.1 (0.7)	2.5 (0.8)
HDL cholesterol, mmol/L	0.9 (0.3)	1.0 (0.2)	1.0 (0.2)	1.0 (0.2)	1.0 (0.2)	0.9 (0.2)	1.0 (0.2)
Triglycerides, mmol/L	1.7 (0.9)	2.0 (0.9)	1.8 (0.7)	1.6 (0.6)	1.4 (0.6)	1.2 (0.5)	1.6 (0.7)
Apolipoprotein B, g/L	1.0 (0.3)	1.0 (0.3)	1.0 (0.2)	0.9 (0.2)	0.9 (0.2)	0.8 (0.2)	0.9 (0.2)
Apolipoprotein A1, g/L	1.1 (0.2)	1.3 (0.2)	1.3 (0.2)	1.2 (0.2)	1.2 (0.2)	1.1 (0.2)	1.2 (0.2)
Albumin, g/L	31.0 (5.9)	36.3 (5.5)	38.5 (3.9)	38.1 (4.0)	37.7 (4.3)	35.1 (5.6)	37.8 (4.3)
Glycoprotein A, mmol/L	1.0 (0.2)	1.0 (0.2)	0.9 (0.1)	0.9 (0.1)	0.8 (0.1)	0.8 (0.1)	0.9 (0.1)

Values are mean (standard deviation), n (column %), or median (interquartile range). Analyses exclude participants with previously diagnosed chronic diseases (ischemic heart disease, cerebrovascular, cirrhosis, cancer, or emphysema) at recruitment (except diabetes and CKI), missing data on NMR-measured creatinine or any analysis covariate (sex, district of residence, educational level, smoking status, alcohol intake, weight, height, waist circumference, hip circumference, diabetes status, or HbA_1__c_), and those with uncertain follow-up.

Abbreviations: BMI, body mass index; CKI, chronic kidney insufficiency; DBP, diastolic blood pressure; eGFR, estimated glomerular filtration rate; HbA_1c_, glycated hemoglobin; HDL, high-density lipoprotein; LDL, low-density lipoprotein; NMR, nuclear magnetic resonance; SBP, systolic blood pressure.

a Measured using the Nightingale Health Ltd NMR platform (to convert creatinine from μmol/L to mg/dL, multiply by 0.01131).

b eGFR calculated using Chronic Kidney Disease Epidemiology Collaboration 2009 equation with NMR-measured creatinine.

c No previously diagnosed diabetes, but HbA_1c_ ≥6.5%.

Compared with participants with higher eGFR, those with lower eGFR tended to be older and were more likely to be men but were less likely to be residents of Coyoacán (the more affluent district), be university or high school educated, or be current smokers or drinkers (Table 1). They also had lower body mass index and higher systolic and diastolic blood pressure and were considerably more likely to have previously diagnosed diabetes and to be taking medications. Concentrations of low-density lipoprotein cholesterol, triglycerides, apolipoprotein B, and the inflammation biomarker glycoprotein A tended to be slightly higher, and those of high-density lipoprotein cholesterol, apolipoprotein A1, and albumin slightly lower, in participants with lower eGFR. Baseline characteristics of participants aged 75-84 years at recruitment are provided in the supplementary material (Table S1). Repeat creatinine measurements were available for 8,170 participants from samples obtained, on average, 15 years later. The correlations between baseline and follow-up creatinine and eGFR levels were 0.49 and 0.56 respectively (Table S2). Among 878 participants with creatinine measured using both nuclear magnetic resonance spectroscopy and standard clinical chemistry, the correlation between estimates was high (*r* = 0.90; Fig S1).

### Associations of eGFR With All-Cause Mortality

During a median follow-up of 20.4 years (interquartile range, 19.5-21.6) among survivors, 12,590 participants died at ages 35-74 years. These deaths included 3,245 from vascular diseases (2,277 cardiac and 700 cerebrovascular deaths), 1,998 from kidney diseases (1,687 CKD deaths), 523 from acute diabetic crises, 1,033 from hepatobiliary diseases, 1,983 from cancer, 2,050 from respiratory diseases, and 783 from infectious diseases (Table S3a). Among the 133,348 participants aged 35-84 years at recruitment, there were 7,165 deaths at ages 75-84 years (Table S3b).

**Figure 1:**
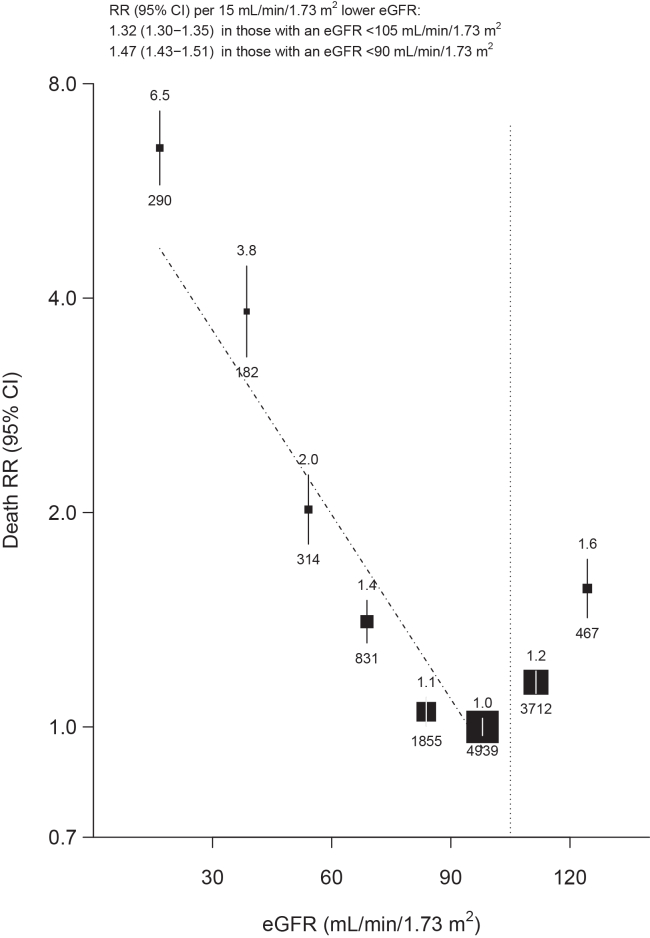
Relevance of estimated glomerular filtration rate (eGFR) to all-cause mortality. Analyses exclude those with self-reported previously diagnosed chronic diseases at recruitment (ischemic heart disease, cerebrovascular, cirrhosis, cancer, or emphysema) except diabetes or chronic kidney disease. The eGFR was derived from nuclear magnetic resonance-measured plasma creatinine using the Chronic Kidney Disease Epidemiology Collaboration 2009 equation. Death rate ratio (RR) estimates are stratified by age at risk (in 5-year ranges) and adjusted for sex, district of residence, educational level, smoking status, alcohol intake, physical activity, adiposity, and diabetes. For each group, the death rate ratio is plotted against the mean eGFR in that group. The vertical lines through each point represent group-specific 95% confidence intervals (CIs), with the area of each square proportional to the amount of statistical information. For each group, the RR is above the square and the number of deaths is below. The vertical line denotes an eGFR of 105 mL/min/1.73 m^2^.

Overall, there was a reverse J-shaped association between eGFR and all-cause mortality (Fig 1). Participants with an eGFR <30 mL/min/1.73 m^2^ had almost 7 times the all-cause mortality rate of those with an eGFR ≥90 to <105 mL/min/1.73 m^2^ (RR, 6.5; 95% CI, 5.7-7.3). In participants with eGFR <105 mL/min/1.73 m^2^, each 15-mL/min/1.73 m^2^ lower eGFR was, on average, associated with 32% higher mortality (RR, 1.32; 95% CI, 1.30-1.35). In participants within the eGFR range <90 mL/min/1.73 m^2^, this inverse relationship was even stronger (RR, 1.47; 95% CI, 1.43-1.51 per 15-mL/min/1.73 m^2^ lower eGFR). In participants with eGFR >105 mL/min/1.73 m^2^, eGFR was positively related to mortality: compared with those with eGFR ≥90 to <105 mL/min/1.73 m^2^, those with eGFR ≥120 mL/min/1.73 m^2^ had 56% higher mortality (RR, 1.56; 95% CI, 1.42-1.73). The inverse association of eGFR with mortality in participants with an eGFR <105 mL/min/1.73 m^2^ was stronger at younger than older ages (although absolute mortality risk increased substantially with age) but, at any given age, was similar in men and women (Fig S2). The associations between eGFR and mortality at different levels of socioeconomic factors, lifestyle characteristics, and adiposity are shown in Figs S3a and S3b. The inclusion of participants with self-reported chronic diseases at baseline (besides diabetes and CKD; Fig S4) or using the CKD-EPI 2021 equation to calculate the eGFR (instead of the 2009 equation; Fig S5) did not materially change the patterns of association seen.

**Figure 2:**
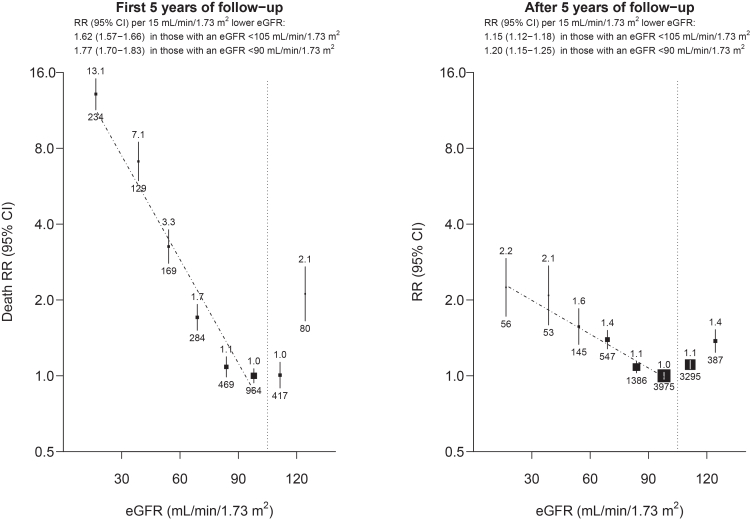
Relevance of estimated glomerular filtration rate (eGFR) to all-cause mortality by follow-up time. Analyses and conventions are as in Fig 1 except the associations of eGFR with mortality are shown separately for deaths occurring during the first 5 years after recruitment (left panel) and for deaths occurring 5 years after recruitment (right panel). CI, confidence interval; RR, death rate ratio.

**Figure 3:**
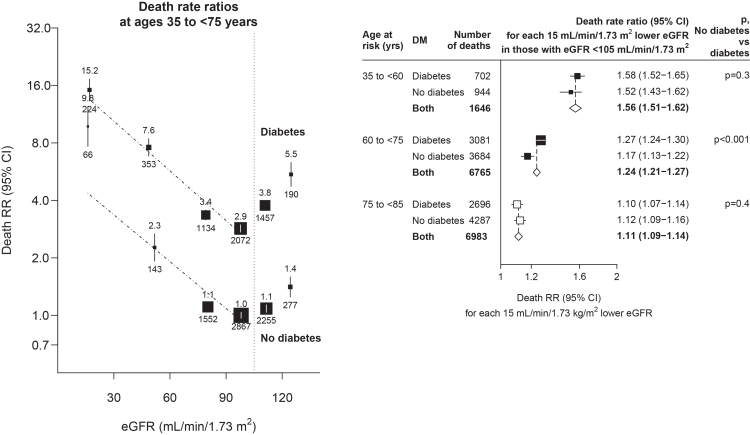
Relevance of eGFR to all-cause mortality by diabetes. Exclusions and conventions are as in Fig 1. Death rate ratio (RR) estimates are stratified by age at risk (in 5-year ranges) and adjusted for sex, district of residence, education level, smoking status, alcohol intake, physical activity, and adiposity. The left panel shows the effects of diabetes (previously diagnosed or undiagnosed) and eGFR (<30, 30-59, 60-89, 90-104, 105-120, and >120 mL/min/1.73 m^2^) plotted against the mean measured eGFR in each group (the reference group is participants without diabetes and with an eGFR 90-104 mL/min/1.73 m^2^). The dashed diagonal lines (left) represent the line of best fit in those with eGFR <105 mL/min/1.73 m^2^ and correspond to an RR per 15-mL/min/1.73 m^2^ lower eGFR of 1.30 (95% CI, 1.26-1.35) in those without diabetes and 1.35 (95% CI, 1.32-1.38) in those with diabetes. At eGFR <90 mL/min/1.73 m^2^, the corresponding RRs per 15-mL/min/1.73 m^2^ lower eGFR were 1.50 (95% CI, 1.43-1.57) in those without diabetes and 1.42 (95% CI, 1.38-1.47) in those with diabetes. The panel on the right shows RRs by age and diabetes status per 15-mL/min/1.73 m^2^ lower eGFR in those with eGFR <105 mL/min/1.73 m^2^. The RRs per 15-mL/min/1.73 m^2^ lower eGFR for the age-at-risk groups of 35-59 years, 60-74 years, and 75-84 years in those with eGFR <90 mL/min/1.73 m^2^ were 1.72 (95% CI, 1.64-1.81), 1.37 (95% CI, 1.33-1.41), and 1.16 (95% CI, 1.13-1.20), respectively. CI, confidence interval; DM, diabetes mellitus; eGFR, estimated glomerular filtration rate.

Subdividing deaths at ages 35-74 years by period of follow-up, the association of eGFR with mortality was much stronger for deaths occurring during the first 5 years of follow-up (RR, 1.62; 95% CI, 1.57-1.66 per 15-mL/min/1.73 m^2^ lower eGFR in the range <105 mL/min/1.73 m^2^) than for deaths occurring in later years (RR, 1.15; 95% CI, 1.12- 1.18) (Fig 2). The association between eGFR and all-cause mortality was similar in shape and strength in individuals with and without diabetes, overall, and at every given age (Fig 3). However, as individuals with diabetes had much higher death rates than those without diabetes, the absolute excess mortality associated with eGFR was greater among those with diabetes. Classifying individuals by both eGFR and diabetes, compared with those without diabetes and eGFR ≥90 to <105 mL/min/1.73 m^2^, the mortality RR at ages 35-74 years for those with diabetes and eGFR <30 mL/min/1.73 m^2^ was 15.2 (95% CI, 13.2-17.6). The positive association of eGFR with mortality observed in individuals with eGFR >105 mL/min/1.73 m^2^ appeared to be stronger in those with than in those without diabetes (Fig 3).

### Associations of eGFR With Cause-Specific Mortality

**Figure 4:**
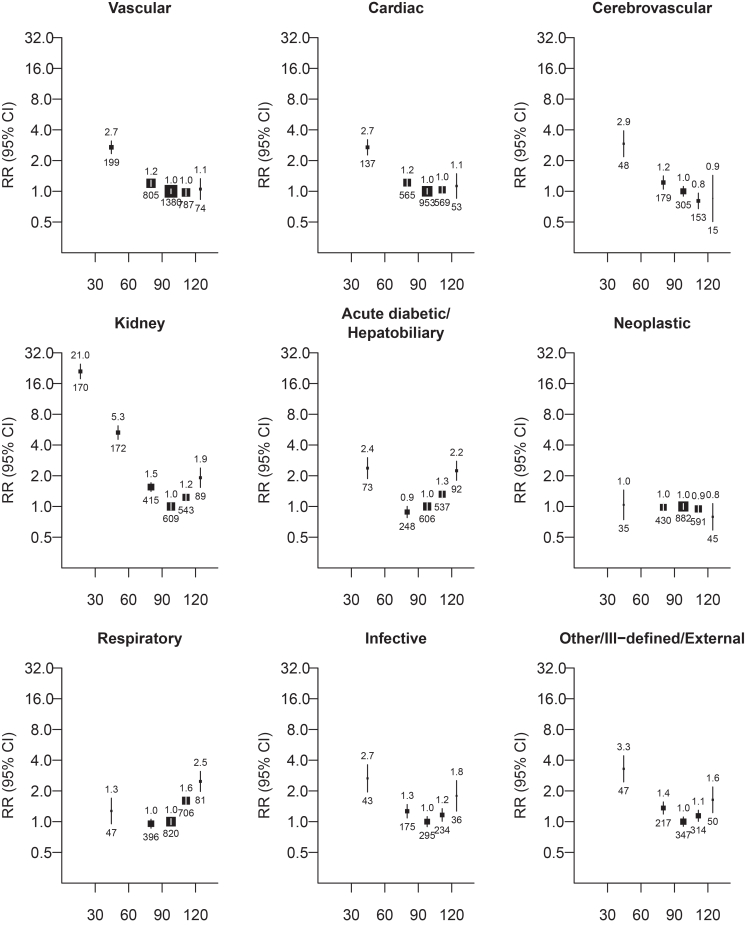
Relevance of eGFR to cause-specific mortality at ages 35-74 years. Analyses, exclusions, and conventions are as in Fig 1. Kidney deaths include those attributed to chronic kidney disease, acute kidney injury, and other kidney causes. The infective endpoint excludes respiratory infections (which are included in the respiratory endpoint). CI, confidence interval; eGFR, estimated glomerular filtration rate; RR, death rate ratio.

Lower eGFR was strongly associated with an increased risk of premature death from kidney, vascular, and infective causes (Fig 4). Compared with those with eGFR ≥90 to <105 mL/min/1.73 m^2^, those with eGFR <30 mL/min/1.73 m^2^ had 21 times the risk of kidney mortality (RR, 21.0; 95% CI, 17.5-25.3), while those with eGFR <60 mL/min/1.73 m^2^ had almost 3 times the risk of cardiac (RR, 2.7; 95% CI, 2.2-3.2), cerebrovascular (RR, 2.9; 95% CI, 2.1-4.0), and infective mortality (RR, 2.7; 95% CI, 1.9-3.7). For participants with eGFR <105 mL/min/1.73 m^2^, each 15-mL/min/1.73 m^2^ lower eGFR was associated with 75% higher kidney mortality (RR, 1.75; 95% CI, 1.69-1.80), 28% higher cardiac mortality (RR 1.28; 95% CI, 1.23-1.34), 30% higher cerebrovascular mortality (RR, 1.30; 95% CI, 1.21-1.40), and 34% higher infective mortality (RR, 1.34; 95% CI, 1.24-1.44) (Table 2). For participants with eGFR <90 mL/min/1.73 m^2^, these associations were even stronger (Table S4). The associations of eGFR with acute diabetic, hepatobiliary, respiratory, and other/ill-defined/external causes were generally weaker and less clear (Fig 4, Table 2, and Fig S6). No association was observed between eGFR and neoplastic mortality. The associations of eGFR with cause-specific mortality were broadly similar in individuals with and without diabetes (Tables S5a and S5b).

**Table 2:** Relevance of eGFR to Cause-Specific Mortality at Ages 35-74 Years, Separately in Those With an eGFR <105 versus ≥105 mL/min/1.73 m^2^

Cause of Death	eGFR <105 mL/min/1.73 m^2^ (74,897 Participants)		eGFR ≥105 mL/min/1.73 m^2^ (51,348 Participants)	
	Deaths	RR (95% CI) per 15 mL/min/1.73 m^2^ Lower eGFR	Deaths	RR (95% CI) per 15 mL/min/1.73 m^2^ Higher eGFR
Cardiac	1,655	1.28 (1.23-1.34)	622	1.26 (1.02-1.56)
Cerebrovascular	532	1.30 (1.21-1.40)	168	1.36 (0.91-2.02)
Other vascular	197	1.22 (1.07-1.39)	71	0.98 (0.50-1.92)
**Subtotal: vascular**	**2,384**	**1.28 (1.24-1.33)**	**861**	**1.26 (1.05-1.51)**
Chronic kidney disease	1,169	1.80 (1.74-1.86)	518	1.79 (1.45-2.19)
Acute kidney injury	52	1.10 (0.83-1.47)	28	2.68 (1.43-5.03)
Other renal disease	145	1.35 (1.17-1.54)	86	2.10 (1.28-3.43)
**Subtotal: kidney disease**	**1,366**	**1.75 (1.69-1.80)**	**632**	**1.87 (1.55-2.24)**
Acute diabetic crisis	329	1.23 (1.13-1.34)	194	1.70 (1.20-2.42)
Hepatobiliary	598	1.04 (0.95-1.15)	435	1.85 (1.50-2.29)
**Subtotal: any of the above (vascular or metabolic)**	**4,677**	**1.44 (1.40-1.47)**	**2,122**	**1.58 (1.42-1.76)**
Neoplastic	1,347	0.99 (0.92-1.05)	636	0.96 (0.76-1.20)
Respiratory	1,263	1.02 (0.96-1.09)	787	1.84 (1.55-2.19)
Infective	513	1.34 (1.24-1.44)	270	1.56 (1.15-2.10)
Other/ill-defined/external	611	1.33 (1.24-1.43)	364	1.56 (1.21-2.00)
**Total: All-causes**	**8,411**	**1.32 (1.30-1.35)**	**4,179**	**1.52 (1.41-1.65)**

Analyses exclude those with chronic disease (ischemic heart disease, cerebrovascular disease, cirrhosis, cancer, or emphysema) at recruitment. Death rate ratio (RR) estimates are stratified by age at risk and adjusted for sex, district of residence, education level, smoking status, alcohol intake, physical activity, adiposity, and diabetes.

Abbreviations: CI, confidence interval; eGFR, estimated glomerular filtration rate.

After further adjusting for baseline measurements of potential mediators and/or markers of frailty (blood pressure, lipids, albumin, and the inflammation biomarker glycoprotein A), the strength of the *inverse* association of lower eGFR with all-cause mortality was reduced by about one-fifth (Figs S7 and S8). For example, compared to participants with eGFR ≥90 to <105 ml/min/1.73 m^2^, the mortality RR for those with eGFR <30 mL/min/1.73 m^2^ was reduced from 6.5 (95% CI, 5.7-7.3) before adjustment (Fig 1) to 4.4 (95% CI, 3.9-5.0) after adjustment (Fig S7). Among those with eGFR ≥105 mL/min/1.73 m^2^, the strength of the *positive* association of higher eGFR with all-cause mortality was also reduced after adjustment for these markers (with the magnitude of this reduction somewhat smaller for those with than without diabetes). The effect of adjustment for potential mediators on associations with particular causes of death is shown in Tables S6a and S6b.

## Discussion

In this study of 130,000 Mexican adults who were followed for over 20 years, lower kidney function measured with creatinine-based eGFR was strongly associated with increased risk of death. Even after adjusting for confounders, including the presence of diabetes and the degree of glycemic control among those with diabetes, the rate of death was approximately 7 times as high in those with eGFR <30 mL/min/1.73 m^2^ as in those with an eGFR ≥90 to <105 mL/min/1.73 m^2^. In participants with eGFR <105 mL/min/1.73 m^2^, each reduction in eGFR of 15 mL/min/1.73 m^2^ was associated with about one-third increase in mortality. The association of eGFR with all-cause mortality was particularly strong for earlier (within the first 5 years) rather than later deaths and was stronger at younger than at older ages, but at any given age, was largely similar irrespective of sex or history of diabetes. However, because the mortality rate was much higher in those with diabetes, the absolute excess mortality associated with lower levels of eGFR was substantially greater for those with than without diabetes.

Decreased eGFR has been consistently associated with mortality and other adverse outcomes in numerous studies.^5^^,^^6^^,^^19^^,^^20^ A recent individual-level meta-analysis of 114 global cohorts involving 27.5 million participants showed a reverse J-shaped association between eGFR and all-cause mortality.^6^ Compared with an eGFR of 90 mL/min/1.73 m^2^, an eGFR of 15 mL/min/1.73 m^2^ was associated with about a doubling to tripling in the risk of all-cause mortality. Decreased eGFR was also associated with higher risk of cardiovascular events, hospitalizations, and death, as well as with higher risk of kidney failure with replacement therapy and of acute kidney injury.^6^ The higher mortality RRs observed in our analyses may be partly explained by differences in the covariates included in the models. Specifically, the meta-analyses were adjusted for urinary albumin-creatinine ratio, blood pressure, total and high-density lipoprotein cholesterol, and the use of antihypertensive medications. Although we were not able to adjust for urinary albumin-creatinine ratio, our analyses that included further adjustment for blood pressure, blood lipids, albumin, and chronic inflammation (which we considered as potential mediators rather than confounders) still found appreciably stronger associations than those reported in the meta-analysis. Although very large, of the 114 cohorts included in the previous meta-analysis of eGFR and adverse outcomes,^6^ only 3 (involving a total of fewer than 3,000 participants) included participants from Latin America. One large-scale nationwide study (not included in the meta-analysis described above) of 758,219 adults with diabetes from Colombia who were followed for 4 years reported that those with an eGFR <30 mL/min/1.73 m^2^ were about 5 times more likely to die of any cause than those with eGFR of ≥90 mL/min/1.73 m^2^,^21^ but the analyses included only limited adjustment for confounders and were not able to explore associations in participants without diabetes.

A probable explanation for the markedly strong associations of low eGFR and kidney mortality (mostly because of CKD) is suboptimal medical care, specifically high levels of glycemia among people with diabetes. In our study, more than one-third of the participants with diagnosed or undiagnosed diabetes at recruitment had an HbA_1c_ >9%. The use of renin angiotensin system blocking agents, which can delay CKD progression in some individuals, was also low. Less than 2 in every 5 participants with eGFR <60 mL/min/1.73 m^2^, which is consistent with CKD, and only 1 in 4 participants with diagnosed diabetes were reportedly using a renin angiotensin system inhibitor at recruitment. By the time of the 2015-2019 resurvey, the use of renin angiotensin system inhibitors had increased only moderately (to about half of those with eGFR <60 mL/min/1.73 m^2^ and about two-fifths of those with diabetes).^11^ Beyond CKD management, it is likely that limited access to kidney replacement therapy, which is not universally available in Mexico,^22^^,^^23^ explains the exceedingly high kidney mortality rates observed in those with an eGFR <60 mL/min/1.73 m^2^. (Indeed, in this group of individuals, 44% of all the deaths observed before age 75 years were because of kidney causes.)

A striking finding from the present analysis is the observation that the association between low eGFR and mortality was substantially stronger for deaths in the first few years of follow-up than for those in subsequent years. This may reflect the ‘horse-racing’ effect,^24^ which describes the general phenomenon whereby the true value of a numerical quantity will tend to be correlated with the true rate of change in that quantity. In the current setting, it may be that the particularly high excess mortality associated with low eGFR in the first few years of follow-up predominantly reflects the risk associated with low eGFR because of recent deterioration in kidney function before recruitment, while the later more modest excess reflects the risk associated with long-term reduced but stable eGFR. Similar to other studies,^6^^,^^20^ we found a positive association between eGFR and all-cause mortality among those with an eGFR >105 mL/min/1.73 m^2^. The excess mortality risk associated with very high eGFR may reflect an increase associated with low creatinine production as a consequence of reductions in muscle mass secondary to illness, malnutrition, or aging (rather than increased risk because of high creatinine clearance). By contrast, the excess risk may reflect kidney hyperfiltration (a compensatory abnormally high GFR, which is commonly observed in early-stage diabetic nephropathy). These hypotheses are supported, at least in part, by our observation that adjusting for potential markers of frailty (including total cholesterol and albumin) weakened the strength of the association of mortality with eGFR >105 mL/min/1.73 m^2^ to a somewhat lesser extent for those with diabetes (where hyperfiltration may be common) than for those without diabetes.

The key strength of the present study is the availability of both a large sample size and prolonged follow-up in a previously understudied population. We adjusted for confounders, excluded participants with pre-existing disease (other than diabetes or kidney disease) and performed a range of sensitivity analyses. However, our study does have some limitations. As we lacked baseline urinary samples, we were not able to evaluate the presence of albuminuria, a well-established risk factor for adverse outcomes that is independent of eGFR.^5^^,^^6^^,^^19^ Thus, we were not able to identify individuals with early stages of CKD (ie, those with an apparently healthy eGFR but with albuminuria) and could not evaluate the extent to which albuminuria was related to mortality. Consequently, if anything, our findings may underestimate the importance of CKD on mortality in this population. We did not have longitudinal measurements of creatinine and could not track eGFR variation or identify the progression or incidence of CKD. We also lacked information on diabetes subtypes and on renal replacement therapy at baseline. In addition, our study of participants from 2 districts of Mexico City is not representative of the entirety of Mexico. However, prospective studies of nonrepresentative cohorts of individuals can still provide reliable evidence about the associations of risk factors with disease that are widely generalizable.^25^ As in most other observational studies, we were unable to control for measurement error (in the exposure or covariates) or for residual confounding. Finally, an absence of information on nonfatal outcomes meant that we could not study CKD incidence, and our results directly apply only to causes of death.

In Mexico, CKD represents a disproportionately high economic burden for the health care system, primarily because of the costs of kidney replacement therapy.^26^ Widening access to hemodialysis or kidney transplantation will undoubtedly improve the quality of life of those with end-stage kidney disease and should be pursued. Nevertheless, early identification and effective management of diabetes is likely to have the largest effect in reducing the burden of CKD in Mexico. For those with diabetes, obesity, and other CKD risk factors, a meaningful reduction of CKD incidence and progression, of cardiovascular outcomes, and of premature mortality will most likely be achieved by increasing use of inexpensive drugs such as renin angiotensin system inhibitors and statins and, when affordable generics become available, by rapidly expanding the use of newer cardio- and renoprotective therapeutic options, including sodium-glucose co-transporter-2 inhibitors^27^^,^^28^ and glucagon-like peptide-1 receptor agonists.^29^^,^^30^

In this middle-income country, where overweight and obesity are very common, and diabetes is frequent and often with high levels of glycemia, decreased kidney function resulted in a substantially increased risk of premature mortality, with the largest absolute excess risk observed in those with diabetes. Optimizing prevention and care of CKD, particularly in individuals with diabetes, will reduce premature mortality in Mexico and should be a focus of disease-prevention policies.

## Supplementary Materials

Supplementary File (PDF) Figure S1-S8; Table S1-S6; Item S1
