# Supplementary material for: Kidney Function and Mortality in Mexico: Prospective Study of 130,000 Adults
Source: Kidney Med. 2026 May 12;8(7):101398. doi: 10.1016/j.xkme.2026.101398 (PMC13265878; doi:10.1016/j.xkme.2026.101398)
Supplement: Supplementary File (PDF) — Figure S1-S8; Table S1-S6; Item S1 [file mmc1.pdf]

Kidney function and mortality in Mexico: prospective study of 130 000 adults

Supplementary Appendix, Table of Contents

Supplementary Figures

		Pg
S1.	Clinical chemistry versus NMR spectroscopy measured creatinine	2
	Relevance of eGFR to all-cause mortality:	
	S2. By age and sex	3
	S3. At ages 35 to <75 years by levels of confounders, in those with:	
	a. eGFR <105 mL/min/1.73m^2	4
	b. eGFR <90 mL/min/1.73m^2	5
	S4. By age and history of diabetes including those with prior chronic disease at recruitment	6
	S5. At ages 35 to <75 years using the CKD-EPI 2021 equation	7
S6.	Relevance of eGFR to mortality at ages 35 to <75 years due to kidney, hepatobiliary, acute diabetes, and respiratory causes	8
	Impact of <u>adjustment</u> for blood pressure, lipids and other biomarkers on:	
S7.	The estimated relevance of eGFR to all-cause mortality at ages 35 to <75	9
S8.	The estimated relevance of eGFR to all-cause mortality by age and history of diabetes	10

Supplementary Tables

S1.	Baseline characteristics of 7 103 participants without prior chronic disease (except diabetes or CKD) aged 75-84 at recruitment, overall and by baseline eGFR	11
S2.	Kidney function at baseline and at resurvey (~15 years later) among 8170 survivors who took part in the resurvey	12
S3.	Number of deaths by underlying cause (ICD-10 code)	
	a. At ages 35 to <75 years	13
	b. At ages 75 to <85 years	15
	Relevance of eGFR to cause-specific mortality at ages 35 to <75 years:	
	S4. In those with eGFR <90 versus ≥90 mL/min/1.73m²	16
	S5. In those with versus without diabetes and:	
	a. eGFR <105 mL/min/1.73m^2	17
	b. eGFR <90 mL/min/1.73m^2	18
S6.	Effect of adjustment for blood pressure, lipids, and other biomarkers on the estimated relevance of eGFR to cause-specific mortality at ages 35 to <75 years:	
	a. In those with an eGFR <105 mL/min/1.73m^2	19
	b. In those with an eGFR <90 mL/min/1.73m^2	20
**Supplementary Methods: Item S1**		21

Supplementary Figure S1: Correlation of plasma creatinine measured with clinical chemistry and with NMR spectroscopy among 878 participants included in the main analyses.

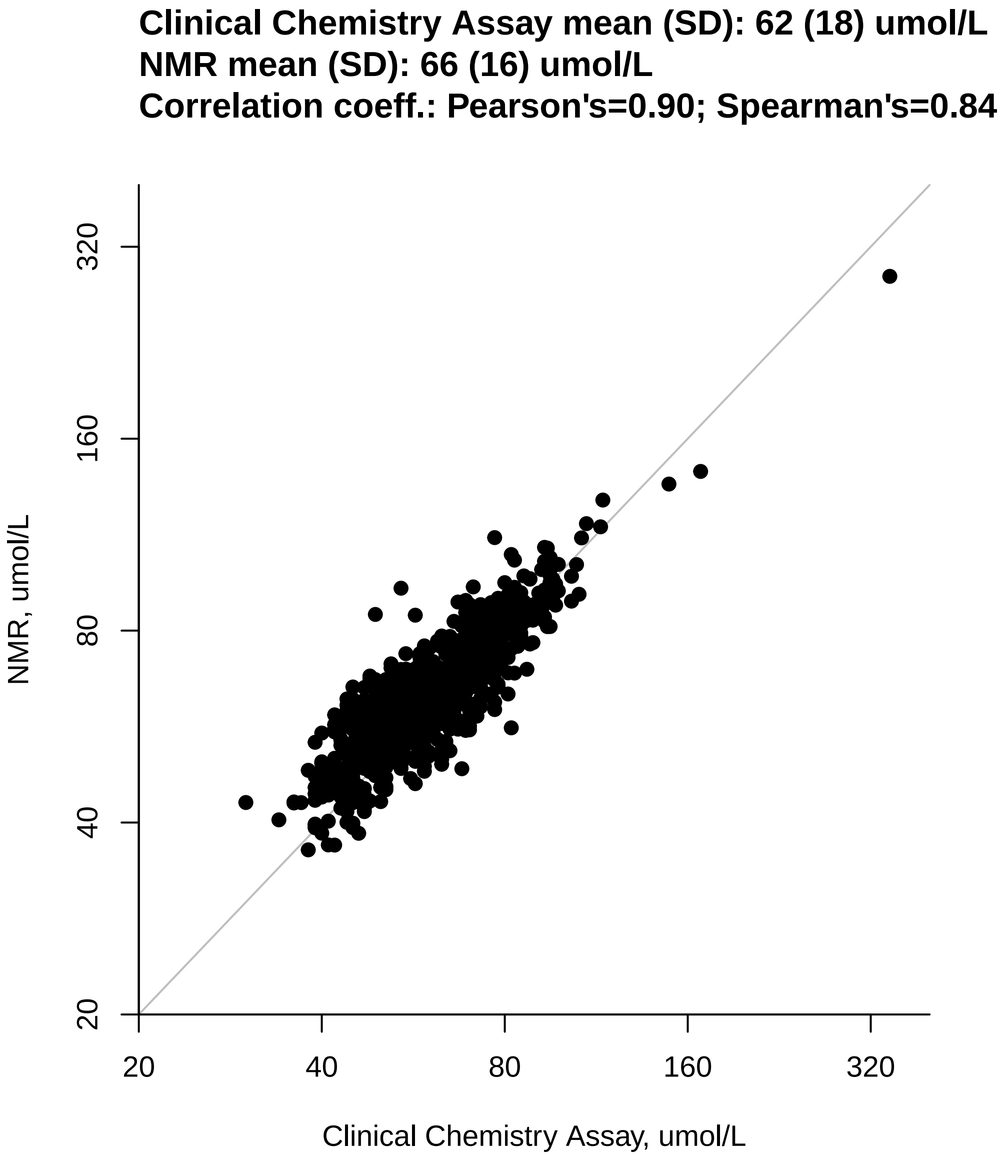

The Pearson's and Spearman's correlation coefficients of log−creatinine were 0.87 and 0.84, respectively.

Supplementary Figure S2: Relevance of eGFR to ALL−CAUSE mortality, by age and sex

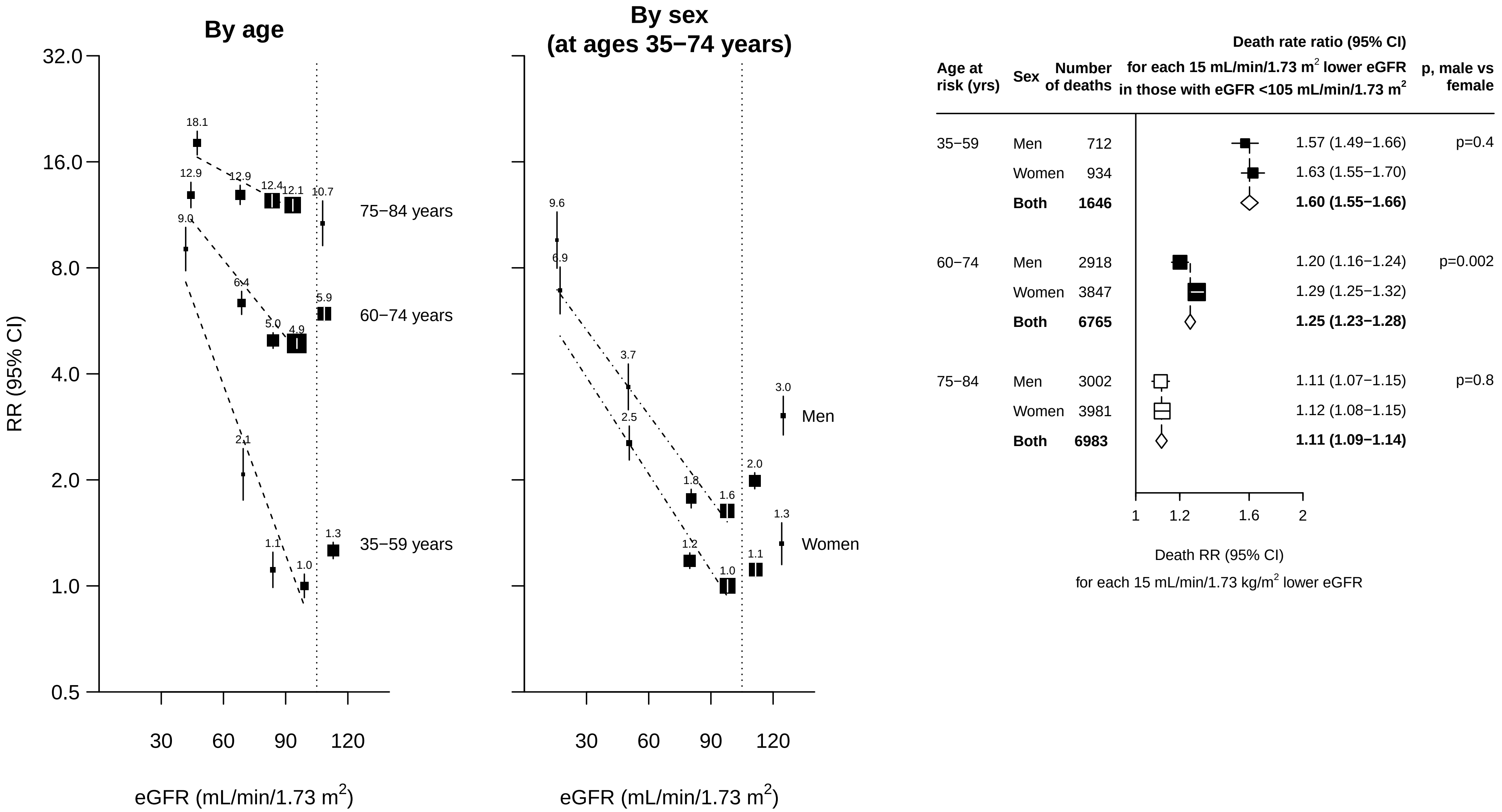

Analyses and conventions as for Figure 1, but now subdivided by age−at−risk and sex. Below 105 mL/min/1.73 m^2, the death RRs (95% CI) at ages 35−74 years per 15 mL/min/1.73 m^2 lower eGFR were 1.28 (1.25−1.32) in men and 1.35 (1.32−1.39) in women. Below 90 mL/min/1.73 m^2, the death RRs at ages 35−74 years per 15 mL/min/1.73 m^2 lower eGFR were 1.44 (1.38−1.50) in men and 1.50 (1.45−1.55) in women and the corresponding death RRs for the age−at−risk groups of 35−59 years, 60−74 years, and 75−84 years were 1.77 (1.68−1.87), 1.39 (1.34−1.43), and 1.16 (1.12−1.19), respectively. A total of 133,348 participants (126,245 aged 35 to <75 years and 7,103 aged 75 to <85 years at baseline) contributed to the analyses presented in this figure.

Supplementary Figure S3a: Relevance of eGFR to mortality at ages 35−74 years by levels of confounders, in those with an eGFR <105 mL/min/1.73 m^2

Variable	Number of deaths	All−cause mortality rate ratio per 15 mL/min/1.73 m^2 lower eGFR, RR (95% CI)	Heterogeneity/ trend
**District**			
Coyoacan	2809	1.28 (1.24−1.33)	$\chi^2_1 = 5.1$
Iztapalapa	5602	1.34 (1.31−1.37)	p=0.02
**Education level**			
University/College	832	1.24 (1.16−1.34)	$\chi^2_3 = 3.3$
High School	1588	1.33 (1.27−1.40)	p=0.4
Elementary	4746	1.33 (1.29−1.36)	
Other	1245	1.34 (1.28−1.40)	
**Smoking**			
Never	3704	1.33 (1.30−1.37)	$\chi^2_4 = 8.8$
Former	1885	1.35 (1.30−1.40)	p=0.07
<Daily	839	1.28 (1.20−1.37)	
Daily <10 cig/day	1228	1.33 (1.26−1.41)	
Daily 10+ cig/day	755	1.21 (1.13−1.30)	
**Alcohol**			
Never	1588	1.40 (1.35−1.45)	$\chi^2_4 = 17.2$
Former	1582	1.33 (1.28−1.37)	p=0.002
Up to 3 times/mo	4652	1.29 (1.25−1.32)	
Up to 2 times/wk	382	1.27 (1.13−1.43)	
3 or more times/wk	207	1.12 (0.95−1.33)	
**Physical activity**			
None	1588	1.40 (1.35−1.45)	$\chi^2_2 = 13.3$
Up to 2 times/wk	1582	1.33 (1.28−1.37)	p=0.001
3 or more times/wk	4652	1.29 (1.25−1.32)	
**Weight**			
I (mean: 56.2kg)	2112	1.36 (1.31−1.40)	$\chi^2_1 = 11.6$
II	1857	1.35 (1.30−1.40)	p<0.001
III	2078	1.27 (1.22−1.32)	
IV (mean: 87.3kg)	2364	1.26 (1.21−1.31)	
**Height**			
I (mean: 148cm)	2312	1.33 (1.29−1.38)	$\chi^2_1 = 1.0$
II	2254	1.28 (1.23−1.33)	p=0.3
III	2059	1.34 (1.29−1.39)	
IV (mean: 164cm)	1786	1.36 (1.30−1.42)	
**Waist circumference**			
I (mean: 81cm)	1601	1.34 (1.29−1.39)	$\chi^2_1 = 3.8$
II	1807	1.35 (1.30−1.41)	p=0.05
III	2206	1.30 (1.26−1.35)	
IV (mean: 109cm)	2797	1.29 (1.24−1.33)	
**Hip circumference**			
I (mean: 93cm)	2154	1.36 (1.32−1.40)	$\chi^2_1 = 7.5$
II	1788	1.31 (1.26−1.36)	p=0.006
III	2017	1.31 (1.25−1.36)	
IV (mean: 118cm)	2452	1.27 (1.22−1.32)	
**Diabetes**			
No diabetes	4628	1.24 (1.20−1.28)	$\chi^2_4 = 49.5$
Undiagnosed diabetes	658	1.14 (1.04−1.26)	p<0.001
Dx diabetes, HbA1c<9%	1328	1.41 (1.37−1.45)	
Dx diabetes, 9 ≤ HbA1c<11%	828	1.34 (1.27−1.41)	
Dx diabetes, HbA1c ≥ 11%	969	1.22 (1.16−1.29)	
**Overall**	**8411**	**1.32 (1.30−1.35)**	

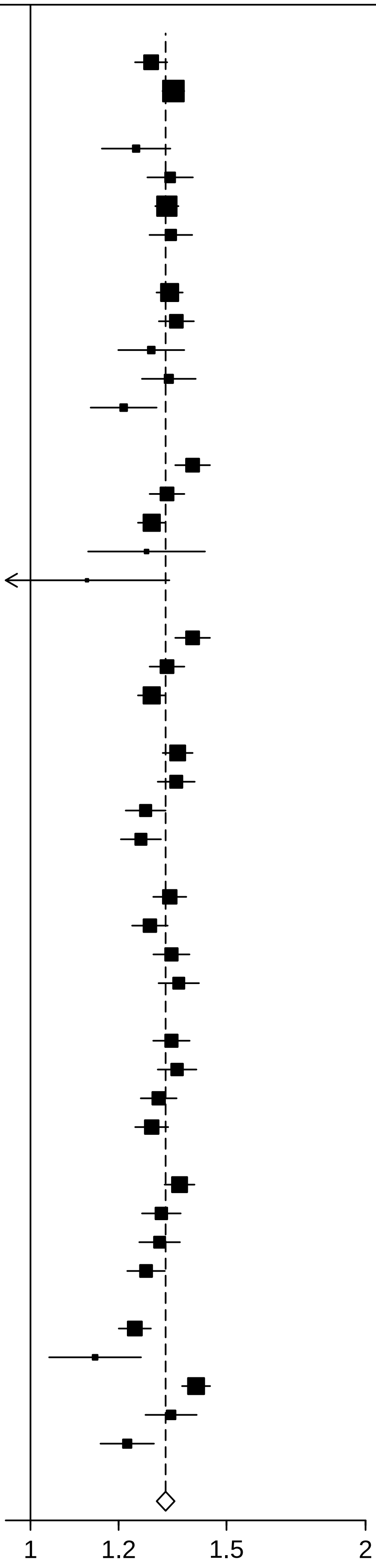

Exclusions and analyses as for Table 2, with the exception that RRs are now estimated separately by levels of each confounder shown (with mutual adjustment for the other shown confounders). A total of 74,897 participants aged 35 to <75 years and with an eGFR <105 mL/min/1.73 m^2 at baseline contributed to the analyses presented in this figure.

Supplementary Figure S3b: Relevance of eGFR to mortality at ages 35−74 years by levels of confounders, in those with an eGFR <90 mL/min/1.73 m^2

Variable	Number of deaths	All−cause mortality rate ratio per 15 mL/min/1.73 m^2 lower eGFR, RR (95% CI)	Heterogeneity/ trend
**District**			
Coyoacan	1059	1.42 (1.35−1.49)	$\chi^2_1 = 3.1$
Iztapalapa	2413	1.50 (1.45−1.55)	p=0.08
**Education level**			
University/College	315	1.55 (1.40−1.72)	$\chi^2_3 = 6.3$
High School	656	1.54 (1.43−1.64)	p=0.1
Elementary	1928	1.47 (1.42−1.52)	
Other	573	1.38 (1.30−1.47)	
**Smoking**			
Never	1532	1.47 (1.42−1.53)	$\chi^2_4 = 5.7$
Former	857	1.48 (1.40−1.56)	p=0.2
<Daily	295	1.54 (1.39−1.70)	
Daily <10 cig/day	486	1.55 (1.43−1.68)	
Daily 10+ cig/day	302	1.34 (1.21−1.48)	
**Alcohol**			
Never	746	1.47 (1.40−1.55)	$\chi^2_4 = 2.7$
Former	708	1.43 (1.36−1.51)	p=0.6
Up to 3 times/mo	1834	1.48 (1.42−1.54)	
Up to 2 times/wk	125	1.66 (1.35−2.04)	
3 or more times/wk	59	1.62 (1.22−2.15)	
**Physical activity**			
None	746	1.47 (1.40−1.55)	$\chi^2_2 = 1.0$
Up to 2 times/wk	708	1.43 (1.36−1.51)	p=0.6
3 or more times/wk	1834	1.48 (1.42−1.54)	
**Weight**			
I (mean: 56kg)	923	1.41 (1.35−1.48)	$\chi^2_1 = 1.3$
II	777	1.51 (1.43−1.60)	p=0.3
III	827	1.46 (1.38−1.55)	
IV (mean: 87.2kg)	945	1.48 (1.39−1.57)	
**Height**			
I (mean: 147cm)	917	1.51 (1.44−1.59)	$\chi^2_1 = 0.4$
II	884	1.40 (1.33−1.48)	p=0.6
III	886	1.46 (1.38−1.54)	
IV (mean: 164cm)	785	1.56 (1.46−1.65)	
**Waist circumference**			
I (mean: 81cm)	665	1.38 (1.30−1.46)	$\chi^2_1 = 2.7$
II	745	1.51 (1.43−1.61)	p=0.1
III	911	1.46 (1.38−1.54)	
IV (mean: 109cm)	1151	1.49 (1.42−1.57)	
**Hip circumference**			
I (mean: 94cm)	929	1.42 (1.35−1.48)	$\chi^2_1 = 2.3$
II	742	1.45 (1.37−1.53)	p=0.1
III	823	1.52 (1.43−1.62)	
IV (mean: 118cm)	978	1.48 (1.40−1.56)	
**Diabetes**			
No diabetes	1761	1.50 (1.43−1.57)	$\chi^2_4 = 7.1$
Undiagnosed diabetes	213	1.42 (1.21−1.66)	p=0.1
Dx diabetes, HbA1c<9%	757	1.46 (1.39−1.52)	
Dx diabetes, $9 \leq$ HbA1c<11%	375	1.40 (1.29−1.51)	
Dx diabetes, HbA1c $\geq$ 11%	366	1.34 (1.22−1.46)	
**Overall**	**3472**	**1.47 (1.43−1.51)**	

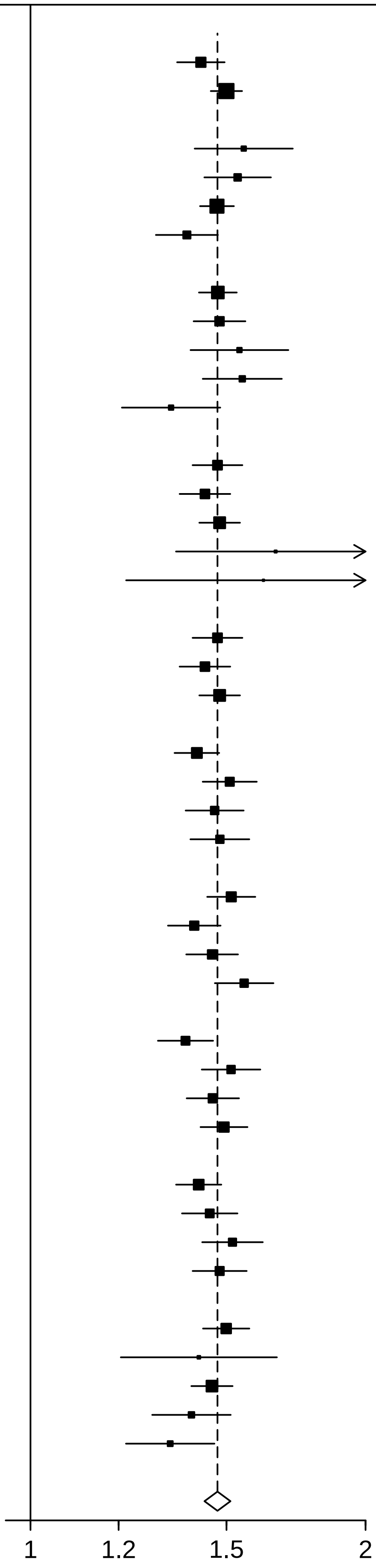

Exclusions and analyses as for Table 2, with the exception that RRs are now estimated separately by levels of each confounder shown (with mutual adjustment for the other shown confounders). A total of 28,529 participants aged 35 to <75 years and with an eGFR <90 mL/min/1.73 m^2 at baseline contributed to the analyses presented in this figure.

Supplementary Figure S4: Relevance of eGFR to ALL−CAUSE mortality further including participants WITH PRIOR CHRONIC DISEASE AT RECRUITMENT

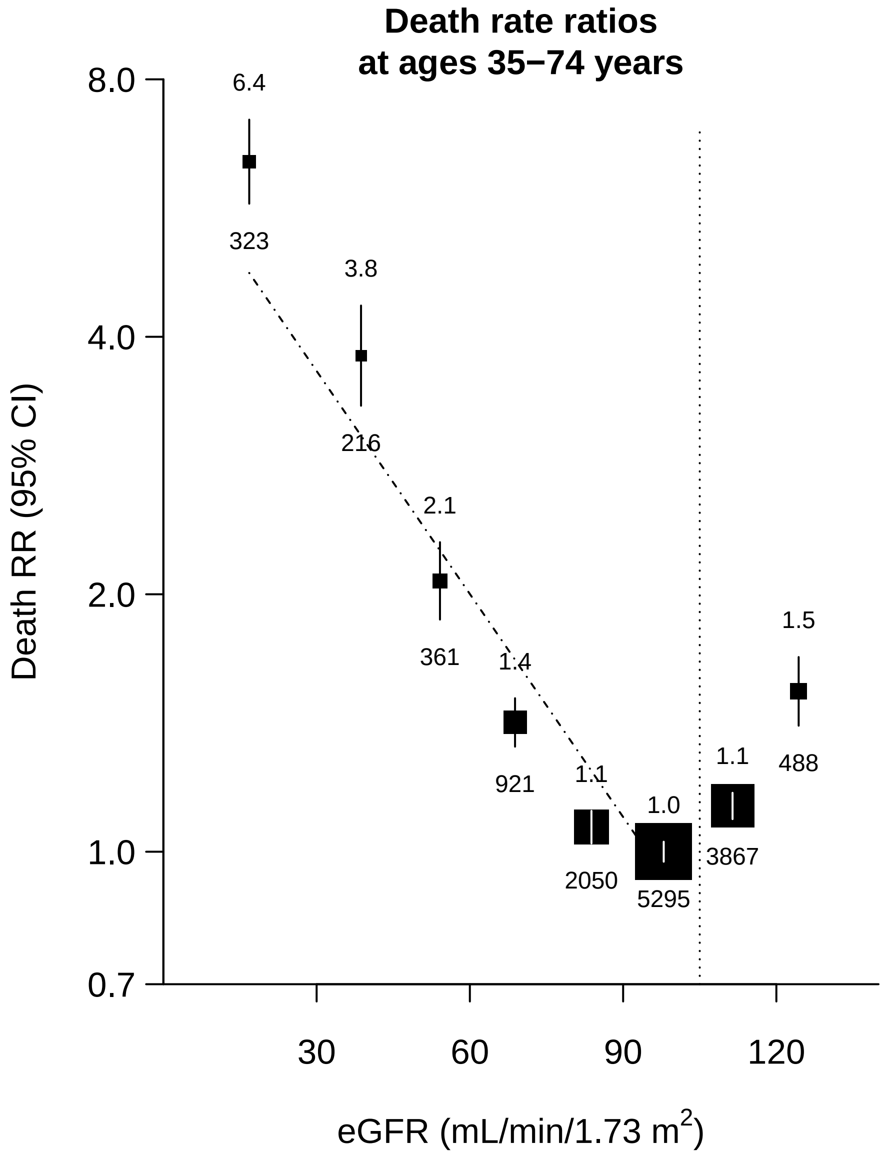

Age at risk (yrs)	DM	Number of deaths	Death rate ratio (95% CI) for each 15 mL/min/1.73 m^2 lower eGFR in those with eGFR <105 mL/min/1.73 m^2	p, No diabetes vs diabetes
35−59	Diabetes	747	1.58 (1.51−1.64)	p=0.4
	No diabetes	1024	1.53 (1.44−1.62)	
	**Both**	**1771**	**1.56 (1.51−1.61)**	
60−74	Diabetes	3362	1.28 (1.25−1.31)	p=0.003
	No diabetes	4033	1.20 (1.15−1.24)	
	**Both**	**7395**	**1.25 (1.23−1.28)**	
75−84	Diabetes	2972	1.10 (1.07−1.13)	p=0.05
	No diabetes	4728	1.15 (1.11−1.18)	
	**Both**	**7700**	**1.12 (1.10−1.15)**	

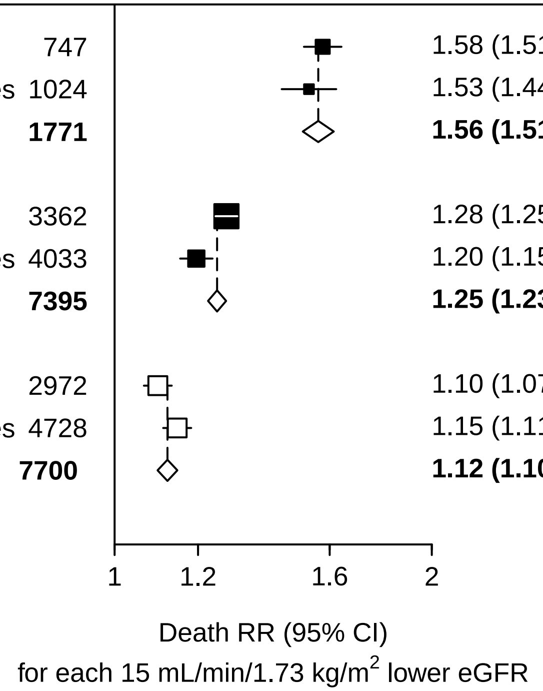

Analyses and conventions as for Figure 1 (left panel) and Figure 2 (right panel) except those with previously diagnosed chronic diseases where further included in the analyses. Below 105 mL/min/1.73 m^2, the death RR (95% CI) at ages 35−74 years per 15 mL/min/1.73 m^2 lower eGFR was 1.33 (1.30−1.35). Below 90 mL/min/1.73 m^2, the death RRs at ages 35−74 years per 15 mL/min/1.73 m^2 lower eGFR was 1.46 (1.43−1.50) and the corresponding death RRs for the age−at−risk groups of 35−59 years, 60−74 years, and 75−84 years were 1.70 (1.62−1.78), 1.37 (1.33−1.41), and 1.17 (1.13−1.20), respectively. 138,819 participants aged 35 to <84 years at baseline contributed to the analyses presented in this figure.

Supplementary Figure S5: Relevance of eGFR to all−cause mortality at ages 35−74 years using the CKD−EPI 2021 equation (instead of the CKD−EPI 2009 equation used in the main analyses)

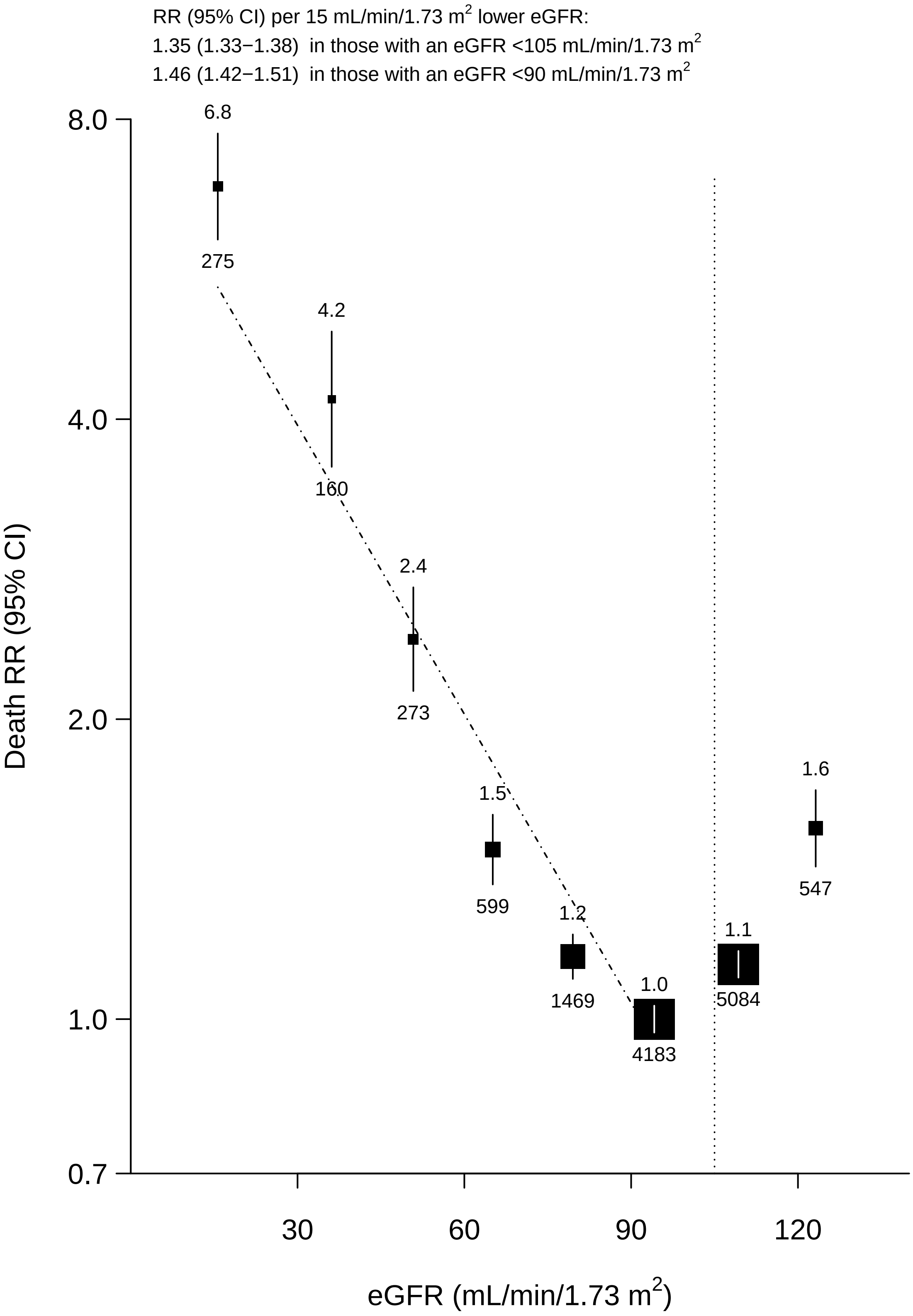

Analyses as per Figure 1 except the CKD−EPI 2021 equation was used to estimate the GFR from baseline creatinine measurements (instead of using the CKD−EPI 2009 equation). 126,245 participants aged 35 to <75 years at baseline contributed to the analyses presented in this figure.

Supplementary Figure S6: Relevance of eGFR to mortality at ages 35−74 years due to kidney, hepatobiliary, acute diabetes, and respiratory causes

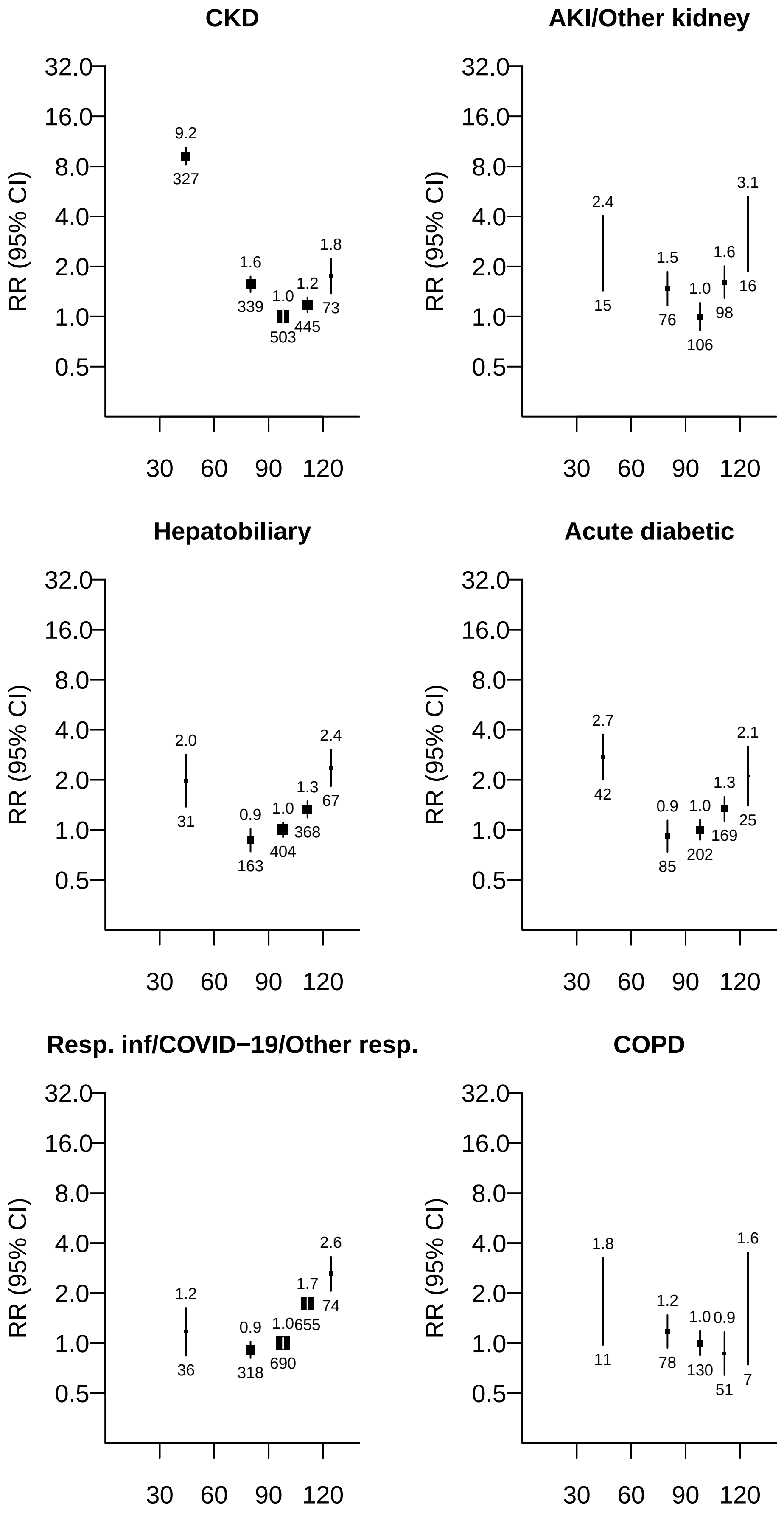

Analyses and exclusions as for Figure 3. The 'Kidney', 'Acute−diabetic/Hepatobiliary', and 'Respiratory' endpoints in Figure 3 were further divided and are shown on the top, middle, and bottom rows, respectively. AKI=Acute kidney injury. 126,245 participants aged 35 to <75 years at baseline contributed to the analyses presented in this figure.

Supplementary Figure S7: Effect of adjustment for blood pressure, lipids and other biomarkers on the relevance of eGFR to all−cause mortality at ages 35−74 years

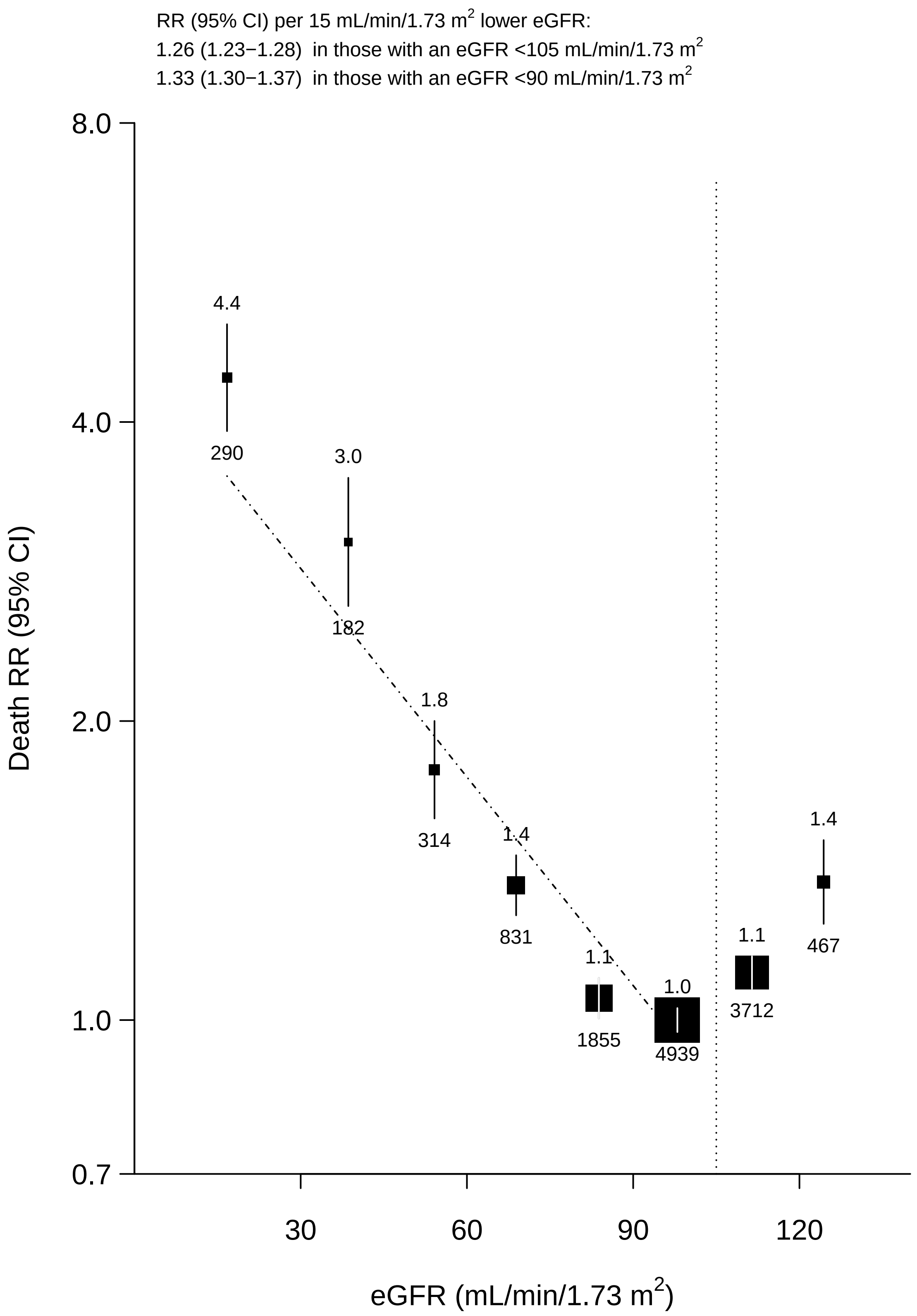

Analyses and conventions as in Figure 1, except models are further adjusted for systolic and diastolic blood pressure, NMR−measured plasma LDL cholesterol, HDL cholesterol, total triglycerides, Apolipoprotein B, Apolipoprotein A1, the inflammation biomarker Glyc−A, and albumin. 126,245 participants aged 35 to <75 years at baseline contributed to the analyses presented in this figure.

Supplementary Figure S8: Effect of adjustment for blood pressure, lipids and other biomarkers on the relevance of eGFR to all−cause mortality by age and diabetes

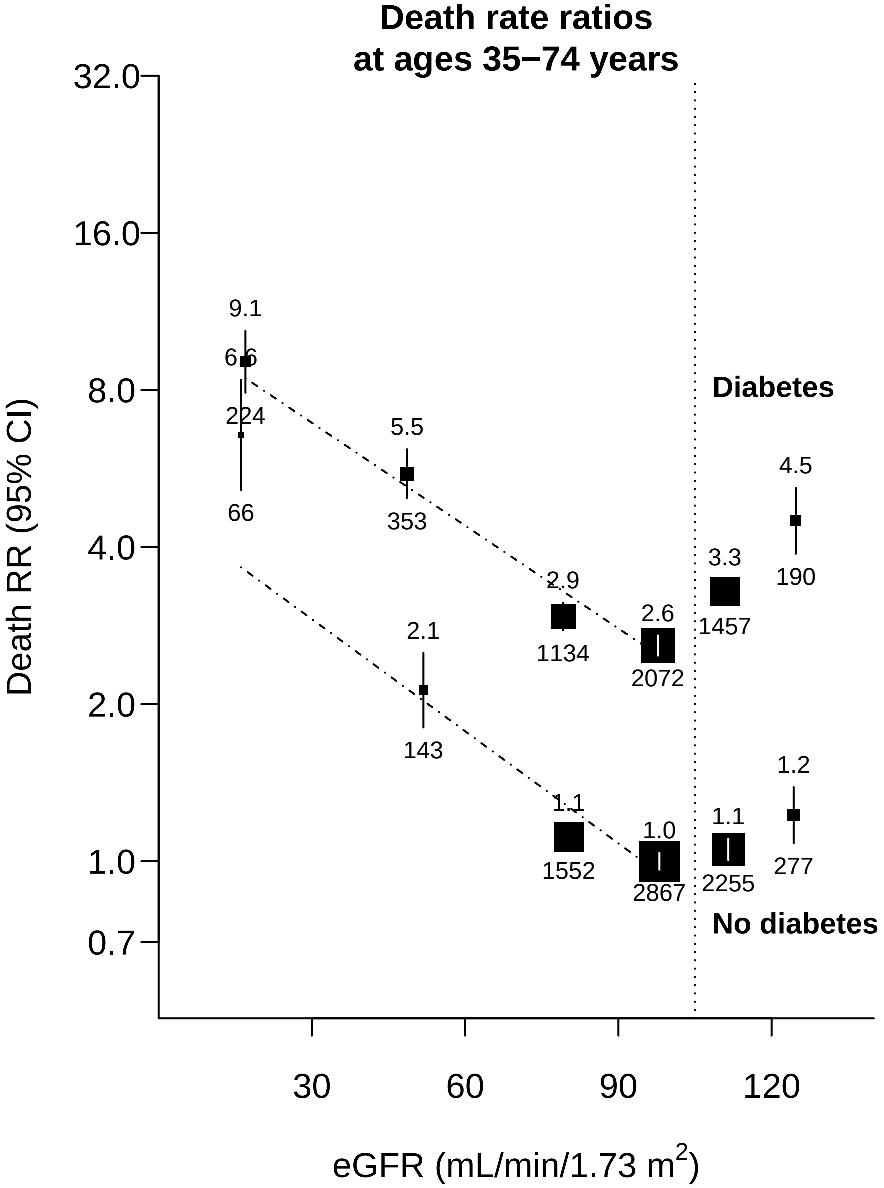

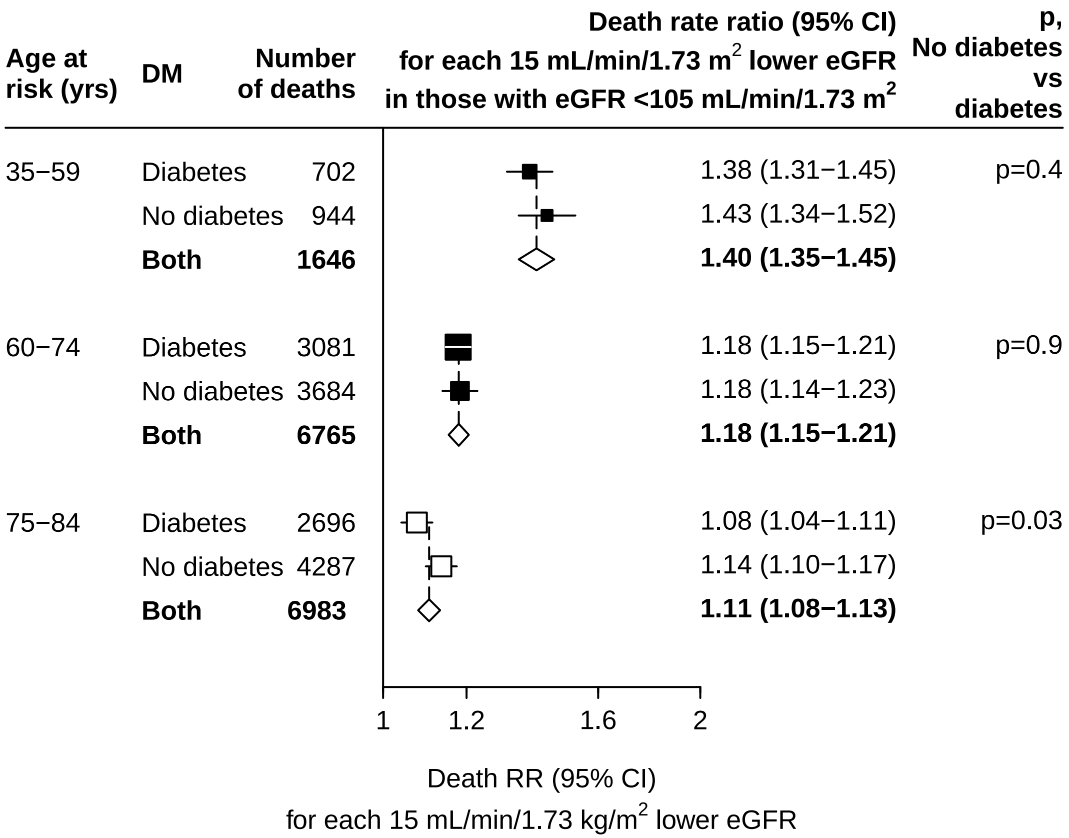

Analyses and conventions as for Figure 2. Below 105 mL/min/1.73 m^2, the death RRs (95% CI) at ages 35−74 years per 15 mL/min/1.73 m^2 lower eGFR were 1.22 (1.19−1.25) in those with and 1.24 (1.20−1.28) in those without diabetes. Below 90 mL/min/1.73 m^2, the death RRs at ages 35−74 years per 15 mL/min/1.73 m^2 lower eGFR were 1.28 (1.24−1.33) in those with and 1.40 (1.33−1.47) in those without diabetes. The corresponding death RRs for the age−at−risk groups of 35−59 years, 60−74 years, and 75−84 years were 1.49 (1.40−1.58), 1.27 (1.23−1.31), and 1.14 (1.10−1.17), respectively. 126,245 participants aged 35 to <75 years at baseline contributed to the analyses presented in this figure.

Supplementary Table S1: Baseline characteristics of 7 103 participants without prior chronic disease (except diabetes or CKD) aged 75-84 years at recruitment, overall and by baseline eGFR

	eGFR, mL/min/1.73m²				Overall
	<30 (n=102)	30 to <60 (n=850)	60 to <90 (n=5 420)	≥90 (n=731)	(n=7 103)
Age, years	79 (3)	79 (3)	79 (3)	78 (2)	79 (3)
Male	33 (32%)	298 (35%)	1994 (37%)	361 (49%)	2686 (38%)
eGFR, mL/min/1.73 m²	22 (15-27)	53 (46-57)	81 (73-85)	93 (91-95)	81 (69-86)
Self-reported CKI	16 (16%)	7 (1%)	31 (1%)	5 (1%)	59 (1%)
**Socio-economic status and lifestyle behaviours**					
Resident of Coyoacán	24 (24%)	263 (31%)	1906 (35%)	308 (42%)	2501 (35%)
University/high school educated	2 (2%)	26 (3%)	208 (4%)	11 (2%)	247 (3%)
Current smoker	13 (13%)	108 (13%)	724 (13%)	132 (18%)	977 (14%)
Current drinker	42 (41%)	347 (41%)	2626 (48%)	365 (50%)	3380 (48%)
**Physical measurements**					
Height, cm	152 (10)	152 (9)	152 (9)	152 (10)	152 (9)
Weight, kg	63 (13)	65 (13)	64 (12)	62 (13)	64 (12)
BMI, kg/m²	27.2 (4.6)	27.9 (4.6)	27.6 (4.6)	26.9 (4.7)	27.6 (4.6)
Waist circumference, cm	97 (11)	98 (12)	97 (11)	96 (11)	97 (11)
Hip circumference, cm	103 (10)	104 (10)	103 (10)	101 (11)	103 (10)
SBP, mmHg	145 (21)	138 (19)	138 (19)	138 (20)	138 (19)
DBP, mmHg	88 (11)	85 (11)	85 (11)	85 (11)	85 (11)
**Diabetes and glycosylated hemoglobin**					
Diabetes status					
No diabetes	52 (51%)	575 (68%)	3975 (73%)	510 (70%)	5112 (72%)
Undiagnosed diabetes	2 (2%)	44 (5%)	362 (7%)	50 (7%)	458 (6%)
Diagnosed diabetes	48 (47%)	231 (27%)	1083 (20%)	171 (23%)	1533 (22%)
HbA_{1c} <9%	38 (37%)	165 (19%)	761 (14%)	102 (14%)	1066 (15%)
HbA_{1c} 9% to <11%	8 (8%)	43 (5%)	211 (4%)	41 (6%)	303 (4%)
HbA_{1c} ≥11%	2 (2%)	23 (3%)	111 (2%)	28 (4%)	164 (2%)
HbA_{1c}, %	6.4 (1.5)	6.4 (1.6)	6.3 (1.5)	6.4 (1.8)	6.3 (1.6)
**Medication use**					
Any glucose-lowering	39 (38%)	190 (22%)	893 (16%)	138 (19%)	1260 (18%)
Any anti-hypertensive	63 (62%)	382 (45%)	1820 (34%)	193 (26%)	2458 (35%)
Renin-angiotensin system inhibitor	50 (49%)	288 (34%)	1312 (24%)	147 (20%)	1797 (25%)
Any anti-thrombotic	5 (5%)	51 (6%)	278 (5%)	28 (4%)	362 (5%)
Any lipid lowering	0 (0%)	6 (1%)	31 (1%)	4 (1%)	41 (1%)
**Lipids and other biomarkers**					
LDL Cholesterol, mmol/L	2.4 (1.0)	2.5 (0.8)	2.4 (0.8)	2.0 (0.7)	2.4 (0.8)
HDL Cholesterol, mmol/L	1.0 (0.2)	1.0 (0.2)	1.0 (0.2)	1.0 (0.2)	1.0 (0.2)
Triglycerides, mmol/L	1.5 (0.6)	1.7 (0.6)	1.5 (0.6)	1.2 (0.4)	1.5 (0.6)
Apolipoprotein B, g/L	0.9 (0.3)	0.9 (0.2)	0.9 (0.2)	0.8 (0.2)	0.9 (0.2)
Apolipoprotein A1, g/L	1.2 (0.2)	1.2 (0.2)	1.2 (0.2)	1.1 (0.2)	1.2 (0.2)
Albumin, g/L	34.0 (4.9)	36.0 (4.6)	36.4 (4.1)	33.8 (5.1)	36.0 (4.3)
Glycoprotein A, mmol/L	0.9 (0.2)	0.9 (0.1)	0.8 (0.1)	0.8 (0.1)	0.8 (0.1)

Mean (SD), median (IQR), or n (column %) shown. BMI=body-mass index; CKI=chronic kidney insufficiency; DBP=diastolic blood pressure; eGFR=estimated Glomerular Filtration Rate, calculated using CKD-EPI (2009) equation with NMR-measured creatinine; SBP=systolic blood pressure.

Excluding participants with previously diagnosed chronic diseases (ischaemic heart disease, cerebrovascular disease, cirrhosis, cancer, or emphysema) at recruitment (except diabetes and CKD), missing data on NMR-measured creatinine or any analysis covariate (sex, district of residence, educational level, smoking status, alcohol intake, weight, height, waist circumference, hip circumference, diabetes status, or HbA_{1c}), or uncertain follow-up. Only 10 participants had an eGFR between 105 to <120 mL/min/1.73 m² (none had eGFR ≥120 mL/min/1.73 m²).

Supplementary Table S2. Kidney function at baseline and at resurvey subdivided by baseline eGFR

		Baseline (median year 2002)			Resurvey (median year 2017)			
**Baseline eGFR, mL/min/1.73 m²**	**No. participants**	**Age, years**	**Creatinine, umol/L**	**eGFR, mL/min/1.73 m²**	**Age, years**	**Creatinine, umol/L**	**eGFR, mL/min/1.73 m²**	**UACR >3 mg/mmol***
<60	67	64 (11)	109 (30)	53 (8)	79 (10)	141 (138)	56 (25)	9/21 (43%)
60 to <75	324	62 (10)	85 (12)	69 (4)	77 (10)	89 (55)	70 (19)	55/143 (38%)
75 to <90	1145	59 (11)	75 (11)	84 (4)	75 (11)	79 (33)	79 (17)	133/461 (29%)
90 to <105	3117	55 (9)	63 (10)	98 (4)	71 (9)	72 (33)	86 (16)	368/1436 (26%)
105 to <120	3148	44 (6)	56 (9)	111 (4)	60 (7)	67 (39)	96 (15)	284/1479 (19%)
≥120	369	40 (4)	46 (7)	124 (4)	56 (5)	63 (41)	102 (14)	44/172 (26%)
**Overall**	**8 170**	**51 (11)**	**63 (14)**	**101 (14)**	**67 (11)**	**72 (40)**	**88 (18)**	**893/3712 (24%)**

Mean (SD) or n/N (%). The (Spearman) correlation coefficient between baseline and resurvey was 0.49 for creatinine and 0.57 for eGFR.

* 3712 participants had urine taken and uACR measured at resurvey. Of these participants, microalbuminuria (uACR >3 mg/mmol) was recorded for 164 (61%) of 268 with eGFR <60 mL/min/1.73 m², 140 (32%) of 431 with eGFR 60 to <75 mL/min/1.73 m², 212 (23%) of 935 with eGFR 75 to <90 mL/min/1.73 m², 313 (18%) of 1706 with eGFR 90 to <105 mL/min/1.73 m², and 64 (17%) of the 367 with eGFR 105 to <120 mL/min/1.73 m² (only 5 participants had an eGFR ≥120 mL/min/1.73 m² at resurvey).

Supplementary Table S3a. Number of deaths at ages 35-74 years by underlying cause (ICD-10 code)

Cause of death	ICD-10 codes (and number of deaths)
Cardiac (n=2277)	I018 (1), I050 (2), I051 (1), I059 (14), I070 (1), I071 (2), I079 (1), I080 (3), I081 (1), I091 (1), I099 (13), I110 (92), I119 (8), I200 (3), I209 (3), I210 (19), I211 (9), I219 (1667), I220 (2), I221 (1), I229 (1), I249 (31), I251 (35), I252 (1), I258 (9), I259 (76), I270 (6), I272 (3), I279 (7), I301 (1), I319 (1), I330 (7), I340 (3), I348 (1), I350 (11), I351 (1), I358 (1), I38X (7), I420 (9), I429 (1), I442 (6), I443 (2), I460 (1), I461 (1), I469 (8), I471 (2), I472 (2), I489 (4), I48X (5), I490 (4), I499 (6), I500 (50), I501 (11), I509 (68), I515 (1), I517 (1), I518 (4), I519 (9), Q210 (1), Q231 (1), Q238 (1), Q248 (1), R570 (32)
Cerebrovascular (n=700)	F019 (2), I600 (2), I608 (1), I609 (77), I61 (1), I610 (1), I612 (1), I613 (1), I614 (2), I615 (2), I618 (1), I619 (206), I620 (8), I629 (4), I633 (2), I634 (15), I635 (3), I638 (2), I639 (47), I64X (99), I669 (5), I671 (5), I672 (1), I674 (3), I678 (79), I679 (94), I690 (1), I693 (7), I694 (5), I698 (23)
Other vascular (n=268)	E115 (34), E145 (28), I260 (2), I269 (73), I710 (4), I712 (1), I713 (4), I718 (3), I719 (1), I729 (2), I731 (1), I739 (3), I740 (1), I741 (1), I743 (1), I771 (13), I776 (1), I802 (6), I803 (1), I822 (1), I828 (1), I829 (3), I830 (1), I839 (1), I872 (5), I879 (1), I890 (1), I99X (4), K550 (62), K551 (1), K552 (1), K559 (5), K761 (1)
Chronic kidney disease (n=1687)	E102 (11), E112 (832), E122 (1), E142 (323), I120 (108), I129 (2), I130 (3), I131 (1), I132 (26), N039 (24), N049 (1), N059 (8), N119 (1), N12X (9), N142 (1), N180 (11), N185 (33), N189 (224), N19X (62), N281 (1), N289 (3), Q619 (1), Y841 (1)
Acute kidney injury (n=80)	N002 (1), N009 (7), N179 (72)
Other kidney (n=231)	N10X (2), N151 (13), N200 (7), N201 (1), N300 (1), N309 (1), N390 (206)
Acute diabetic (n=523)	E100 (2), E101 (4), E110 (154), E111 (172), E140 (99), E141 (84), E162 (8)
Hepatobiliary (n=1033)	B169 (2), B171 (22), B181 (2), B182 (12), B189 (1), B190 (2), B199 (2), D136 (1), I81X (1), I850 (16), I859 (5), K563 (1), K701 (17), K702 (1), K703 (154), K704 (24), K709 (18), K711 (2), K716 (1), K720 (12), K721 (64), K729 (194), K739 (2), K742 (1), K743 (2), K745 (2), K746 (270), K750 (13), K754 (3), K759 (2), K764 (1), K766 (10), K767 (12), K768 (1), K769 (13), K800 (4), K801 (9), K802 (4), K803 (3), K804 (1), K805 (2), K810 (15), K811 (4), K819 (5), K822 (2), K829 (5), K830 (14), K831 (2), K839 (1), K851 (1), K852 (3), K858 (7), K859 (35), K85X (23), K861 (3), K868 (3), Q447 (1)
Neoplastic (n=1983)	C029 (11), C049 (1), C069 (1), C07X (2), C089 (1), C109 (3), C119 (1), C139 (1), C140 (4), C142 (1), C159 (15), C160 (1), C169 (190), C170 (11), C179 (1), C182 (3), C184 (1), C187 (2), C189 (110), C19X (4), C20X (17), C211 (1), C220 (52), C221 (24), C229 (102), C23X (26), C240 (11), C241 (9), C248 (3), C249 (15), C250 (24), C259 (95), C260 (2), C269 (3), C319 (1), C329 (11), C33X (1), C349 (138), C37X (1), C382 (1), C383 (1), C384 (1), C412 (2), C414 (1), C419 (6), C435 (1), C437 (1), C438 (1), C439 (11), C444 (1), C445 (1), C447 (1), C449 (5), C450 (1), C451 (2), C457 (1), C459 (4), C469 (1), C480 (9), C482 (4), C492 (3), C495 (1), C499 (10), C509 (179), C519 (3), C52X (1), C530 (1), C539 (102), C541 (21), C549 (1), C55X (11), C56X (103), C609 (2), C61X (63), C629 (1), C64X (76), C679 (20), C680 (2), C689 (1), C694 (1), C709 (2), C710 (13), C711 (1), C718 (2), C719 (38), C720 (1), C729 (1), C73X (23), C741 (1), C749 (1), C759 (1), C760 (5), C762 (2), C763 (2), C764 (1), C765 (2), C780 (6), C786 (5), C787 (8), C788 (2), C793 (2), C794 (2), C795 (1), C796 (1), C798 (3), C800 (26), C809 (20), C80X (3), C817 (1), C819 (8), C829 (1), C830 (1), C833 (12), C838 (1), C839 (2), C844 (1), C845 (1), C851 (1), C857 (1), C859 (44), C900 (38), C901 (1), C902 (1), C910 (25), C911 (1), C919 (1), C920 (27), C921 (5), C927 (3), C929 (3), C950 (2), C959 (3), D371 (5), D372 (1), D374 (5), D376 (6), D377 (2), D380 (2), D381 (6), D383 (2), D391 (2), D397 (1), D410 (3), D419 (1), D429 (1), D430 (13), D432 (2), D449 (1), D486 (1), D487 (9), D489 (3)
Respiratory (n=2050)*	A162 (7), A165 (1), A169 (2), B206 (2), B440 (1), B441 (1), B909 (1), E848 (1), J069 (1), J09 (3), J09X (1), J100 (2), J110 (1), J111 (1), J129 (4), J151 (3), J157 (2), J159 (35), J180 (48), J181 (29), J182 (2), J188 (1), J189 (586), J209 (2), J22X (17), J348 (1), J391 (1), J42X (13), J439 (26), J440 (77), J441 (2), J448 (5), J449 (154), J459 (11), J46X (2), J47X (2), J60X (1), J64X (5), J677 (1), J679 (2), J680 (1), J690 (4), J80X (4), J81X (4), J841 (74), J848 (1), J849 (8), J850 (1), J852 (2), J869 (8), J90X (4), J939 (1), J942 (2), J960 (5), J961 (1), J969 (3), J980 (1), J981 (1), J984 (14), J985 (4), J988 (6), J989 (2), Q311 (1), U071 (539), U072 (301), U099 (1)
Infective (n=783)	A047 (2), A060 (1), A090 (17), A099 (31), A09X (12), A170 (1), A180 (1), A181 (2), A182 (1), A199 (3), A415 (1), A419 (182), A483 (1), A498 (1), A810 (2), A86X (2), B200 (1), B201 (1), B207 (4), B208 (7), B210 (1), B212 (1), B218 (1), B227 (2), B238 (4), B24X (4), B259 (1), B451 (1), B462 (1), B465 (2), B690 (1), B948 (2), B99X (1), G009 (3), G039 (4), G042 (2), G049 (8), G060 (2), H440 (1), H669 (1), K052 (1), K222 (1), K223 (1), K228 (1), K251 (3), K254 (8), K255 (10), K256 (2), K259 (5), K264 (6), K265 (2), K269 (2), K274 (3), K275 (1), K290 (5), K291 (2), K292 (1), K295 (4), K297 (1), K318 (6), K352 (5), K353 (3), K358 (5), K359 (2), K37X (1), K572 (1), K578 (6), K579 (12), K610 (3), K611 (1), K612 (1), K650 (24), K658 (1), K659 (52), K920 (27), K921 (3), K922 (109), L021 (4), L022 (6), L023 (2), L024 (1), L031 (5), L038 (1), L039 (3), L089 (43), L899 (8), L89X (3), L905 (1), L984 (6), M009 (1), M600 (2), M725 (1), M726 (17), M798 (21), M869 (2), N410 (1), N498 (8), N499 (1), N719 (1), N739 (2), N764 (1), R572 (5)

Table continued overleaf.

Supplementary Table S3a. Number of deaths at ages 35-74 years by underlying cause (ICD-10 code)

Cause of death	ICD-10 codes (and number of deaths)
Other/External/Ill-defined** (n=975)	D033 (1), D27X (1), D320 (1), D329 (7), D352 (1), D420 (1), D464 (1), D467 (1), D469 (6), D471 (1), D472 (1), D474 (1), D479 (1), D619 (4), D649 (5), D693 (2), D694 (1), D696 (3), D699 (3), D70X (2), D733 (1), E035 (1), E039 (9), E049 (1), E055 (1), E059 (3), E065 (1), E116 (2), E119 (5), E129 (1), E146 (3), E149 (7), E249 (2), E279 (1), E43X (1), E440 (1), E660 (1), E725 (1), E835 (1), E86X (4), E870 (1), E872 (12), E875 (2), E876 (1), E878 (1), E889 (2), F03X (6), F09X (1), F102 (7), F209 (1), G10X (6), G121 (1), G122 (18), G20X (7), G231 (1), G300 (1), G309 (3), G310 (1), G35X (2), G379 (1), G409 (10), G419 (2), G589 (1), G610 (4), G709 (1), G710 (2), G809 (1), G822 (1), G919 (4), G931 (11), G934 (5), G935 (1), G936 (2), G958 (1), I10X (3), K088 (1), K102 (1), K137 (1), K389 (1), K403 (1), K404 (1), K409 (1), K419 (1), K420 (4), K429 (2), K430 (1), K439 (1), K440 (1), K460 (3), K461 (1), K469 (4), K513 (1), K529 (6), K560 (2), K562 (1), K566 (38), K593 (5), K630 (1), K631 (22), K632 (4), K635 (1), K638 (2), K639 (2), K918 (1), K931 (1), L100 (1), L109 (1), L511 (1), L512 (1), L921 (1), L958 (1), L988 (1), M050 (1), M068 (1), M069 (15), M100 (1), M109 (1), M139 (1), M311 (1), M313 (1), M319 (1), M321 (4), M329 (2), M340 (1), M349 (1), M469 (1), M623 (4), M993 (1), N40X (7), N948 (1), O720 (1), R040 (1), R100 (2), R11X (1), R190 (1), R571 (16), R579 (2), R58X (2), R64X (1), R688 (21), R69X (1), R99X (193), S729 (2), T07X (1), T874 (1), V011 (1), V029 (1), V041 (1), V049 (1), V051 (1), V093 (2), V099 (66), V182 (1), V439 (1), V489 (1), V494 (1), V496 (1), V499 (10), V580 (1), V785 (1), V878 (6), V892 (10), V899 (4), W018 (1), W060 (1), W100 (13), W104 (1), W108 (2), W126 (1), W130 (9), W134 (3), W135 (1), W138 (1), W139 (2), W170 (4), W172 (1), W174 (1), W178 (2), W179 (1), W18 (1), W180 (4), W181 (1), W184 (2), W188 (1), W190 (10), W194 (2), W195 (1), W199 (3), W200 (1), W206 (1), W250 (1), W314 (1), W340 (1), W704 (1), W744 (1), W748 (3), W769 (1), W780 (1), W789 (1), W849 (2), W878 (2), X09 (1), X090 (3), X094 (1), X099 (1), X219 (1), X360 (1), X459 (1), X470 (1), X590 (2), X594 (1), X598 (1), X599 (27), X640 (1), X680 (1), X700 (5), X702 (1), X708 (1), X740 (3), X780 (3), X800 (1), X840 (1), X910 (1), X912 (1), X914 (2), X950 (3), X954 (16), X955 (1), X959 (1), X990 (7), X994 (4), X999 (2), Y044 (2), Y084 (1), Y094 (1), Y099 (1), Y159 (1), Y200 (1), Y240 (1), Y244 (3), Y245 (1), Y248 (1), Y249 (2), Y260 (2), Y280 (1), Y330 (1), Y334 (1), Y338 (1), Y340 (5), Y344 (6), Y346 (1), Y348 (2), Y349 (14), Y405 (1), Y579 (2), Y838 (1), Y839 (4), Y846 (1)

*Includes deaths from respiratory infection.

** Ill-defined deaths include ICD-10 codes R00-R99 (Symptoms, signs and abnormal clinical and laboratory findings, not elsewhere classified) plus ICD-10 codes E86 (Volume depletion), E870 (Hyperosmolality and hypernatraemia), E872 (Acidosis), E873 (Alkalosis) and E874 (Mixed disorder of acid-base balance).

Supplementary Table S3b. Number of deaths at ages 75-84 years by underlying cause (ICD-10 code)

Cause of death	ICD-10 codes (and number of deaths)
Cardiac (n=1786)	I050 (1), I059 (5), I060 (1), I071 (1), I080 (2), I091 (1), I099 (4), I110 (90), I119 (18), I209 (2), I210 (6), I211 (7), I212 (1), I213 (2), I219 (1294), I229 (1), I249 (23), I251 (34), I255 (1), I258 (5), I259 (72), I270 (1), I272 (1), I279 (8), I289 (1), I318 (1), I330 (1), I350 (6), I358 (3), I38X (3), I420 (7), I441 (1), I442 (5), I443 (4), I469 (3), I471 (1), I472 (1), I489 (4), I48X (6), I490 (7), I499 (12), I500 (33), I501 (4), I509 (78), I518 (1), I519 (2), R570 (21)
Cerebrovascular (n=545)	F019 (6), I608 (1), I609 (19), I614 (1), I615 (1), I619 (113), I620 (2), I629 (3), I633 (1), I634 (11), I635 (8), I638 (1), I639 (64), I64X (74), I669 (3), I671 (2), I674 (3), I678 (83), I679 (104), I691 (1), I693 (5), I694 (8), I698 (31)
Other vascular (n=171)	E105 (1), E115 (11), E145 (15), I260 (1), I269 (35), I709 (1), I713 (2), I714 (8), I719 (1), I720 (1), I739 (2), I771 (12), I779 (1), I802 (1), I828 (1), I829 (2), I872 (1), I888 (1), I99X (4), K550 (57), K552 (2), K559 (11)
Chronic kidney disease (n=578)	E102 (1), E112 (247), E142 (96), I120 (78), I129 (1), I130 (1), I131 (2), I132 (10), I139 (1), N039 (10), N059 (1), N180 (3), N185 (8), N189 (86), N19X (28), N289 (3), Q612 (1), Q613 (1)
Acute kidney injury (n=64)	N009 (3), N179 (61)
Other kidney (n=157)	N10X (1), N133 (2), N151 (2), N202 (1), N308 (1), N390 (150)
Acute diabetic (n=310)	E100 (1), E110 (95), E111 (115), E121 (1), E140 (52), E141 (40), E162 (6)
Hepatobiliary (n=329)	B162 (1), B171 (4), B182 (10), D134 (1), I864 (1), K701 (1), K703 (31), K704 (2), K709 (4), K720 (6), K721 (25), K729 (58), K745 (1), K746 (92), K750 (4), K760 (1), K766 (6), K767 (3), K769 (3), K800 (3), K801 (3), K803 (6), K810 (12), K811 (6), K818 (1), K819 (3), K820 (2), K823 (1), K828 (1), K829 (3), K830 (13), K831 (1), K851 (1), K859 (18), K863 (1)
Neoplastic (n=894)	C01X (1), C029 (4), C050 (1), C059 (1), C069 (1), C07X (4), C099 (1), C109 (1), C140 (2), C159 (6), C160 (1), C169 (75), C170 (4), C180 (1), C181 (1), C183 (1), C189 (44), C19X (3), C20X (6), C210 (1), C220 (23), C221 (15), C227 (1), C229 (42), C23X (20), C240 (3), C241 (2), C249 (9), C250 (9), C258 (1), C259 (51), C261 (2), C311 (1), C329 (6), C33X (1), C340 (1), C348 (1), C349 (74), C383 (1), C384 (2), C402 (2), C412 (1), C419 (1), C437 (1), C439 (7), C443 (1), C444 (1), C449 (1), C457 (1), C459 (3), C480 (3), C482 (1), C493 (1), C499 (8), C509 (32), C519 (3), C52X (1), C539 (23), C541 (4), C55X (1), C56X (23), C609 (1), C61X (106), C629 (1), C64X (21), C65X (1), C679 (15), C680 (1), C710 (5), C715 (1), C719 (9), C73X (12), C751 (2), C753 (1), C759 (2), C760 (6), C762 (6), C763 (2), C765 (1), C767 (1), C780 (1), C786 (3), C787 (9), C798 (4), C800 (12), C809 (12), C80X (1), C811 (1), C819 (5), C829 (1), C831 (1), C833 (4), C845 (2), C859 (21), C900 (17), C910 (4), C920 (10), C921 (1), C922 (1), C927 (1), C929 (2), C930 (1), C950 (1), C959 (2), C97X (1), D371 (2), D374 (2), D376 (9), D377 (9), D381 (3), D414 (1), D430 (6), D431 (2), D432 (1), D440 (1), D483 (1), D487 (2)
Respiratory (n=1289)*	A162 (2), E840 (1), J069 (1), J100 (1), J129 (2), J150 (1), J152 (1), J159 (18), J180 (56), J181 (28), J182 (3), J188 (1), J189 (390), J209 (1), J22X (13), J40X (2), J42X (19), J439 (29), J440 (141), J441 (5), J448 (7), J449 (157), J459 (7), J46X (1), J47X (1), J64X (4), J65X (1), J684 (1), J80X (3), J81X (6), J841 (40), J849 (2), J869 (2), J90X (2), J949 (1), J960 (4), J961 (2), J969 (2), J981 (1), J984 (10), J988 (10), J989 (1), U071 (188), U072 (121)
Infective (n=508)	A090 (11), A099 (21), A09X (4), A170 (1), A418 (1), A419 (92), B238 (1), B377 (1), B699 (1), G009 (2), G039 (1), G049 (2), K20X (2), K220 (1), K251 (2), K252 (1), K254 (12), K255 (6), K259 (9), K264 (6), K265 (6), K266 (1), K269 (2), K274 (2), K275 (2), K290 (3), K291 (1), K295 (3), K296 (1), K297 (6), K311 (1), K316 (1), K318 (7), K319 (2), K352 (2), K353 (2), K358 (1), K37X (3), K573 (2), K578 (7), K579 (12), K610 (3), K650 (10), K658 (4), K659 (30), K920 (22), K921 (2), K922 (104), L020 (1), L021 (1), L022 (2), L023 (2), L029 (1), L031 (3), L032 (1), L039 (2), L088 (1), L089 (21), L899 (9), L89X (2), L905 (6), L984 (10), M009 (1), M726 (6), M798 (14), N498 (3), N719 (2), R572 (1)
Other/External/Ill-defined** (n=533)	D181 (1), D320 (3), D352 (1), D369 (1), D464 (1), D469 (4), D471 (1), D589 (1), D619 (3), D62X (1), D649 (2), D65X (2), D691 (1), D695 (1), D699 (1), D70X (1), E039 (2), E055 (1), E059 (1), E116 (2), E119 (4), E146 (2), E149 (3), E271 (1), E43X (3), E46X (3), E86X (2), E870 (1), E871 (1), E872 (4), E875 (2), E876 (1), E878 (4), F03X (10), F329 (2), G112 (1), G122 (1), G20X (20), G219 (1), G301 (4), G309 (5), G312 (1), G35X (1), G409 (4), G473 (1), G603 (1), G610 (1), G919 (1), G931 (2), G934 (3), G958 (1), G959 (1), I10X (2), K102 (1), K403 (6), K404 (1), K409 (2), K413 (2), K419 (1), K420 (2), K429 (2), K430 (1), K439 (1), K460 (5), K469 (3), K509 (1), K519 (1), K560 (1), K562 (4), K564 (1), K566 (42), K567 (1), K593 (3), K631 (12), K632 (2), K638 (2), K669 (1), L100 (1), L511 (1), L512 (3), L97X (1), L989 (2), M050 (1), M069 (4), M100 (1), M300 (1), M321 (1), M331 (1), M350 (1), M471 (2), M623 (7), M729 (1), M819 (3), N40X (5), N768 (1), N823 (1), N859 (1), R092 (2), R100 (1), R13X (1), R31X (1), R54X (1), R571 (11), R578 (2), R579 (1), R58X (3), R64X (1), R688 (6), R69X (1), R91X (1), R99X (112), V031 (1), V099 (29), V149 (1), V299 (1), V494 (1), V499 (1), V581 (1), V892 (1), W104 (1), W130 (8), W170 (2), W180 (5), W184 (1), W190 (5), W198 (1), W199 (3), W228 (1), W557 (1), W748 (1), W780 (2), W808 (1), X440 (1), X53 (1), X530 (1), X590 (6), X599 (17), X700 (1), X740 (1), X800 (1), X841 (1), X910 (1), X950 (1), X990 (1), X994 (1), Y018 (1), Y044 (1), Y090 (2), Y239 (1), Y334 (1), Y340 (1), Y344 (4), Y345 (1), Y348 (2), Y349 (3), Y579 (1), Y609 (1), Y834 (1), Y839 (2), Y86X (3)

*Includes deaths from respiratory infection.

** Ill-defined deaths include ICD-10 codes R00-R99 (Symptoms, signs and abnormal clinical and laboratory findings, not elsewhere classified) plus ICD-10 codes E86 (Volume depletion), E870 (Hyperosmolality and hypernatraemia), E872 (Acidosis), E873 (Alkalosis) and E874 (Mixed disorder of acid-base balance).

Table S4: Relevance of estimated glomerular filtration rate to cause-specific mortality at ages 35-74 years, separately in those with eGFR <90 versus ≥90 mL/min/1.73 m²

	eGFR <90 mL/min/1.73 m² (28 529 participants)		eGFR ≥90 mL/min/1.73 m² (97 716 participants)	
**Cause of death**	**Deaths**	**RR (95% CI) per 15 mL/min/1.73 m² lower eGFR**	**Deaths**	**RR (95% CI) per 15 mL/min/1.73 m² higher eGFR**
Cardiac	702	1.38 (1.29 to 1.47)	1575	1.16 (1.05 to 1.29)
Cerebrovascular	227	1.36 (1.22 to 1.51)	473	0.89 (0.74 to 1.08)
Other vascular	75	1.43 (1.19 to 1.73)	193	1.01 (0.75 to 1.37)
**Subtotal: vascular**	**1004**	**1.38 (1.31 to 1.45)**	**2241**	**1.09 (1.00 to 1.19)**
Chronic kidney disease	666	1.94 (1.85 to 2.04)	1021	1.48 (1.31 to 1.67)
Acute kidney injury	20	0.95 (0.56 to 1.61)	60	1.58 (0.96 to 2.61)
Other renal disease	71	1.42 (1.16 to 1.74)	160	2.18 (1.63 to 2.92)
**Subtotal: kidney disease**	**757**	**1.89 (1.80 to 1.98)**	**1241**	**1.56 (1.40 to 1.74)**
Acute diabetic crisis	127	1.45 (1.28 to 1.63)	396	1.68 (1.38 to 2.04)
Hepatobiliary	194	1.21 (1.04 to 1.40)	839	1.55 (1.36 to 1.76)
**Subtotal: any of the above (vascular or metabolic)**	**2082**	**1.59 (1.54 to 1.64)**	**4717**	**1.33 (1.25 to 1.41)**
Neoplastic	465	1.02 (0.91 to 1.15)	1518	0.96 (0.87 to 1.07)
Respiratory	443	1.12 (1.01 to 1.24)	1607	1.80 (1.63 to 1.98)
Infective	218	1.44 (1.29 to 1.60)	565	1.35 (1.14 to 1.59)
Other/Ill-defined/External	264	1.35 (1.21 to 1.51)	711	1.30 (1.12 to 1.50)
**Total: All-causes**	**3472**	**1.47 (1.43 to 1.51)**	**9118**	**1.33 (1.28 to 1.39)**

Analyses exclude those with chronic disease (ischaemic heart disease, cerebrovascular disease, cirrhosis, cancer, or emphysema) at recruitment. Death rate ratio (RR) estimates are stratified by age at risk and adjusted for sex, district of residence, education level, smoking status, alcohol intake, physical activity, adiposity, and diabetes.

Supplementary Table S5a: Relevance of eGFR to cause-specific mortality at ages 35-74 years in those with versus without diabetes (and eGFR <105 mL/min/1.73 m^2)

	No diabetes (59 121 participants)		Diabetes (15 776 participants)		All (74 897 participants)	
**Cause of death**	**Deaths**	**RR (95% CI) per 15 mL/min/1.73 m² lower eGFR**	**Deaths**	**RR (95% CI) per 15 mL/min/1.73 m² lower eGFR**	**Deaths**	**RR (95% CI) per 15 mL/min/1.73 m² lower eGFR**
Cardiac	879	1.26 (1.17 to 1.36)	776	1.27 (1.20 to 1.33)	1655	1.28 (1.23 to 1.34)
Cerebrovascular	284	1.36 (1.21 to 1.54)	248	1.25 (1.14 to 1.37)	532	1.30 (1.21 to 1.40)
Other vascular	92	1.13 (0.88 to 1.45)	77	1.27 (1.08 to 1.48)	197	1.22 (1.07 to 1.39)
**Subtotal: vascular**	**1272**	**1.27 (1.19 to 1.35)**	**1112**	**1.26 (1.21 to 1.32)**	**2384**	**1.28 (1.24 to 1.33)**
Chronic kidney disease	209	2.43 (2.24 to 2.63)	960	1.65 (1.59 to 1.70)	1169	1.80 (1.74 to 1.86)
Acute kidney injury	28	1.12 (0.72 to 1.74)	24	1.06 (0.74 to 1.54)	52	1.10 (0.83 to 1.47)
Other renal disease	68	1.54 (1.23 to 1.92)	77	1.25 (1.06 to 1.48)	145	1.35 (1.17 to 1.54)
**Subtotal: any kidney disease**	**305**	**2.17 (2.02 to 2.33)**	**1061**	**1.62 (1.56 to 1.67)**	**1366**	**1.75 (1.69 to 1.80)**
Acute diabetic crisis	63	1.01 (0.72 to 1.41)	266	1.24 (1.14 to 1.35)	329	1.23 (1.13 to 1.34)
Hepatobiliary	431	1.09 (0.96 to 1.23)	167	0.98 (0.85 to 1.14)	598	1.04 (0.95 to 1.15)
**Subtotal: any of the above (vascular or metabolic)**	**2071**	**1.40 (1.34 to 1.47)**	**2606**	**1.41 (1.38 to 1.44)**	**4677**	**1.44 (1.40 to 1.47)**
Neoplastic	1051	1.05 (0.97 to 1.13)	296	0.86 (0.76 to 0.98)	1347	0.99 (0.92 to 1.05)
Respiratory	831	0.93 (0.85 to 1.02)	432	1.09 (1.01 to 1.19)	1263	1.02 (0.96 to 1.09)
Infective	251	1.32 (1.16 to 1.51)	262	1.32 (1.21 to 1.43)	513	1.34 (1.24 to 1.44)
Other/Ill-defined/External	424	1.39 (1.26 to 1.54)	187	1.27 (1.15 to 1.41)	611	1.33 (1.24 to 1.43)
**Total: All-causes**	**4628**	**1.24 (1.20 to 1.28)**	**3783**	**1.34 (1.31 to 1.36)**	**8411**	**1.32 (1.30 to 1.35)**

Analyses exclude those with chronic disease (ischaemic heart disease, cerebrovascular disease, cirrhosis, cancer, or emphysema) at recruitment and are restricted to those with eGFR <105 mL/min/1.73 m². Death rate ratio (RR) estimates are stratified by age at risk and adjusted for sex, district of residence, education level, smoking status, alcohol intake, physical activity, and adiposity.

Supplementary Table S5b: Relevance of eGFR to cause-specific mortality at ages 35-74 years in those with versus without diabetes (and eGFR <90 mL/min/1.73 m^2)

	No diabetes (21 837 participants)		Diabetes (6692 participants)		All (28 529 participants)	
Cause of death	Deaths	RR (95% CI) per 15 mL/min/1.73 m² lower eGFR	Deaths	RR (95% CI) per 15 mL/min/1.73 m² lower eGFR	Deaths	RR (95% CI) per 15 mL/min/1.73 m² lower eGFR
Cardiac	358	1.41 (1.26 to 1.58)	344	1.32 (1.23 to 1.42)	702	1.38 (1.29 to 1.47)
Cerebrovascular	116	1.58 (1.33 to 1.87)	111	1.28 (1.12 to 1.46)	227	1.36 (1.22 to 1.51)
Other vascular	38	1.38 (0.98 to 1.95)	37	1.41 (1.13 to 1.76)	75	1.43 (1.19 to 1.73)
**Subtotal: vascular**	**512**	**1.45 (1.32 to 1.59)**	**492**	**1.32 (1.24 to 1.40)**	**1004**	**1.38 (1.31 to 1.45)**
Chronic kidney disease	114	2.93 (2.64 to 3.25)	552	1.72 (1.63 to 1.80)	666	1.94 (1.85 to 2.04)
Acute kidney injury	10	0.74 (0.25 to 2.17)	10	1.05 (0.57 to 1.90)	20	0.95 (0.56 to 1.61)
Other renal disease	36	1.55 (1.11 to 2.15)	35	1.42 (1.11 to 1.83)	71	1.42 (1.16 to 1.74)
**Subtotal: any kidney disease**	**160**	**2.57 (2.34 to 2.82)**	**597**	**1.70 (1.62 to 1.78)**	**757**	**1.89 (1.80 to 1.98)**
Acute diabetic crisis	22	1.22 (0.73 to 2.04)	105	1.47 (1.30 to 1.66)	127	1.45 (1.28 to 1.63)
Hepatobiliary	140	1.35 (1.12 to 1.62)	54	1.05 (0.84 to 1.33)	194	1.21 (1.04 to 1.40)
**Subtotal: any of the above (vascular or metabolic)**	**834**	**1.72 (1.63 to 1.83)**	**1248**	**1.51 (1.45 to 1.56)**	**2082**	**1.59 (1.54 to 1.64)**
Neoplastic	373	1.13 (0.99 to 1.30)	92	0.87 (0.69 to 1.09)	465	1.02 (0.91 to 1.15)
Respiratory	284	0.97 (0.81 to 1.16)	159	1.16 (1.02 to 1.31)	443	1.12 (1.01 to 1.24)
Infective	98	1.62 (1.35 to 1.95)	120	1.33 (1.17 to 1.50)	218	1.44 (1.29 to 1.60)
Other/Ill-defined/External	172	1.56 (1.35 to 1.80)	92	1.15 (0.99 to 1.34)	264	1.35 (1.21 to 1.51)
**Total: All-causes**	**1761**	**1.50 (1.43 to 1.57)**	**1711**	**1.42 (1.38 to 1.47)**	**3472**	**1.47 (1.43 to 1.51)**

Analyses exclude those with chronic disease (ischaemic heart disease, cerebrovascular disease, cirrhosis, cancer, or emphysema) at recruitment and are restricted to those with eGFR <105 mL/min/1.73 m². Death rate ratio (RR) estimates are stratified by age at risk and adjusted for sex, district of residence, education level, smoking status, alcohol intake, physical activity, and adiposity.

Supplementary Table S6a: Effect of adjustment for blood pressure, lipids, and other biomarkers on the estimated relevance of eGFR to cause-specific mortality at ages 35-74 years (in those with eGFR <105 mL/min/1.73 m²)

		eGFR <105 mL/min/1.73 m² (74 897 participants)			
		RR (95% CI) per 15 mL/min/1.73 m² lower eGFR			
**Cause of death**	**Deaths**	**Main adjustments**	**Further adjusted for BP**	**Further adjusted for BP and lipids**	**Further adjusted for BP, lipids, albumin and glycoprotein A**
Cardiac	1655	1.28 (1.23 to 1.34)	1.25 (1.20 to 1.31)	1.25 (1.19 to 1.30)	1.20 (1.15 to 1.25)
Cerebrovascular	532	1.30 (1.21 to 1.40)	1.26 (1.17 to 1.35)	1.27 (1.18 to 1.37)	1.21 (1.12 to 1.31)
Other vascular	197	1.22 (1.07 to 1.39)	1.20 (1.06 to 1.37)	1.21 (1.06 to 1.38)	1.16 (1.01 to 1.32)
**Subtotal: vascular**	**2384**	**1.28 (1.24 to 1.33)**	**1.25 (1.21 to 1.29)**	**1.25 (1.21 to 1.30)**	**1.20 (1.15 to 1.24)**
Chronic kidney disease	1169	1.80 (1.74 to 1.86)	1.75 (1.69 to 1.81)	1.74 (1.68 to 1.81)	1.60 (1.54 to 1.66)
Acute kidney injury	52	1.10 (0.83 to 1.47)	1.12 (0.84 to 1.49)	1.12 (0.84 to 1.48)	1.06 (0.80 to 1.41)
Other renal disease	145	1.35 (1.17 to 1.54)	1.36 (1.19 to 1.56)	1.37 (1.19 to 1.57)	1.30 (1.13 to 1.50)
**Subtotal: any kidney disease**	**1366**	**1.75 (1.69 to 1.80)**	**1.71 (1.66 to 1.77)**	**1.70 (1.65 to 1.76)**	**1.57 (1.51 to 1.63)**
Acute diabetic crisis	329	1.23 (1.13 to 1.34)	1.22 (1.12 to 1.33)	1.20 (1.10 to 1.31)	1.16 (1.06 to 1.27)
Hepatobiliary	598	1.04 (0.95 to 1.15)	1.04 (0.95 to 1.14)	1.06 (0.97 to 1.16)	1.09 (1.00 to 1.20)
**Subtotal: any of the above (vascular or metabolic)**	**4677**	**1.44 (1.40 to 1.47)**	**1.41 (1.38 to 1.44)**	**1.40 (1.37 to 1.43)**	**1.33 (1.30 to 1.36)**
Neoplastic	1347	0.99 (0.92 to 1.05)	0.99 (0.92 to 1.05)	1.01 (0.95 to 1.08)	0.99 (0.93 to 1.06)
Respiratory	1263	1.02 (0.96 to 1.09)	1.03 (0.96 to 1.09)	1.01 (0.95 to 1.08)	0.98 (0.92 to 1.05)
Infective	513	1.34 (1.24 to 1.44)	1.33 (1.23 to 1.43)	1.34 (1.24 to 1.44)	1.27 (1.18 to 1.37)
Other/Ill-defined/External	611	1.33 (1.24 to 1.43)	1.32 (1.23 to 1.42)	1.32 (1.23 to 1.42)	1.29 (1.20 to 1.39)
**Total: All-causes**	**8411**	**1.32 (1.30 to 1.35)**	**1.31 (1.28 to 1.33)**	**1.31 (1.28 to 1.33)**	**1.26 (1.23 to 1.28)**

Analyses exclude those with chronic disease (ischaemic heart disease, cerebrovascular disease, cirrhosis, cancer, or emphysema) at recruitment. All death rate ratio (RR) estimates are stratified by age at risk and adjusted for sex, district of residence, education level, smoking status, alcohol intake, physical activity, adiposity, and diabetes.

Supplementary Table S6b: Effect of adjustment for blood pressure, lipids, and other biomarkers on the estimated relevance of eGFR to cause-specific mortality at ages 35-75 years (in those with eGFR <90 mL/min/1.73 m²)

		eGFR <90 mL/min/1.73 m² (28 529 participants)			
		RR (95% CI) per 15 mL/min/1.73 m² lower eGFR			
**Cause of death**	**Deaths**	**Main adjustments**	**Further adjusted for BP**	**Further adjusted for BP and lipids**	**Further adjusted for BP, lipids, albumin and glycoprotein A**
Cardiac	702	1.38 (1.29 to 1.47)	1.34 (1.25 to 1.42)	1.31 (1.22 to 1.39)	1.22 (1.14 to 1.31)
Cerebrovascular	227	1.36 (1.22 to 1.51)	1.29 (1.16 to 1.44)	1.26 (1.13 to 1.41)	1.18 (1.05 to 1.33)
Other vascular	75	1.43 (1.19 to 1.73)	1.42 (1.17 to 1.71)	1.40 (1.15 to 1.70)	1.33 (1.09 to 1.63)
**Subtotal: vascular**	**1004**	**1.38 (1.31 to 1.45)**	**1.33 (1.26 to 1.40)**	**1.30 (1.23 to 1.37)**	**1.22 (1.16 to 1.29)**
Chronic kidney disease	666	1.94 (1.85 to 2.04)	1.89 (1.80 to 1.99)	1.88 (1.78 to 1.98)	1.71 (1.61 to 1.81)
Acute kidney injury	20	0.95 (0.56 to 1.61)	0.97 (0.57 to 1.67)	0.94 (0.56 to 1.59)	0.88 (0.52 to 1.51)
Other renal disease	71	1.42 (1.16 to 1.74)	1.43 (1.17 to 1.76)	1.41 (1.15 to 1.74)	1.36 (1.10 to 1.69)
**Subtotal: any kidney disease**	**757**	**1.89 (1.80 to 1.98)**	**1.85 (1.77 to 1.95)**	**1.83 (1.74 to 1.93)**	**1.67 (1.58 to 1.77)**
Acute diabetic crisis	127	1.45 (1.28 to 1.63)	1.42 (1.25 to 1.61)	1.38 (1.21 to 1.58)	1.39 (1.21 to 1.60)
Hepatobiliary	194	1.21 (1.04 to 1.40)	1.20 (1.04 to 1.39)	1.18 (1.02 to 1.36)	1.16 (1.00 to 1.34)
**Subtotal: any of the above (vascular or metabolic)**	**2082**	**1.59 (1.54 to 1.64)**	**1.55 (1.50 to 1.60)**	**1.51 (1.46 to 1.56)**	**1.41 (1.36 to 1.46)**
Neoplastic	465	1.02 (0.91 to 1.15)	1.02 (0.91 to 1.15)	1.01 (0.90 to 1.14)	0.98 (0.88 to 1.11)
Respiratory	443	1.12 (1.01 to 1.24)	1.12 (1.01 to 1.25)	1.10 (0.99 to 1.22)	1.04 (0.94 to 1.16)
Infective	218	1.44 (1.29 to 1.60)	1.42 (1.28 to 1.59)	1.43 (1.28 to 1.59)	1.33 (1.19 to 1.50)
Other/Ill-defined/External	264	1.35 (1.21 to 1.51)	1.34 (1.20 to 1.50)	1.33 (1.19 to 1.48)	1.29 (1.15 to 1.45)
**Total: All-causes**	**3472**	**1.47 (1.43 to 1.51)**	**1.45 (1.41 to 1.49)**	**1.42 (1.38 to 1.46)**	**1.33 (1.30 to 1.37)**

Analyses exclude those with chronic disease (ischaemic heart disease, cerebrovascular disease, cirrhosis, cancer, or emphysema) at recruitment. All death rate ratio (RR) estimates are stratified by age at risk and adjusted for sex, district of residence, education level, smoking status, alcohol intake, physical activity, adiposity, and diabetes.

SUPPLEMENTARY METHODS: ITEM S1

Study design and participants

Between 1998 and 2004, households in the neighbouring districts of Coyoacán and Iztapalapa in Mexico City were visited and adults aged 35 years or older invited to participate. Of 112,333 households with eligible inhabitants, one or more individuals from 106,059 (95%) households consented to participate, yielding 159,755 participants in total. Ethics approval was obtained from the Mexican Ministry of Health, the Mexican National Council of Science and Technology, and the University of Oxford, UK. All participants provided written informed consent.

Data collection

During household visits trained nurses administered electronic questionnaires which recorded demographics, socio-economic status, lifestyle factors, current medication, and medical history including previously diagnosed diabetes and chronic kidney insufficiency (CKI; there was no internationally standardised definition of CKD until 2002). Physical measurements including blood pressure, weight, height, waist and hip circumference were taken. A non-fasting venous blood sample was collected into an ethylene-diamine-tetra-acetic (EDTA) acid vacutainer and separated into plasma and buffy coat aliquots for long-term storage at -150°C. Glycated haemoglobin (HbA1c) concentrations were measured in buffy coat samples using a validated high-performance liquid chromatography method.[1] To assess the extent to which exposures varied over time, a resurvey of 10 144 surviving participants took place in 2015-2019. The resurvey involved repeat questionnaire data, physical measures and the collection of EDTA blood and, in a subset, spot urine samples. Ethnicity was not recorded at the baseline or resurvey assessments.

assessments.

Assessment of kidney function

Between 2018 and 2024, a nuclear magnetic resonance spectroscopy metabolomics platform[2] was used to quantify 249 circulating biomarkers from plasma samples collected at baseline (n=152 833) and resurvey (n=9657). The majority of samples (~80%) were analysed at Nightingale Health Plc (Helsinki and Kuopio, Finland) and the remainder were analysed with the same protocol validated for use at the Clinical Trial Service Unit's (CTSU) Wolfson Laboratory (Oxford, UK). Of the 249 biomarkers provided by the platform, 37 including creatinine have been clinically validated. In addition, a random sample of ~1000 baseline plasma samples were analysed for standardized clinical chemistry measurements, including isotope dilution mass spectrometry-traceable creatinine. Each participant's eGFR was estimated using the Chronic Kidney Disease Epidemiology Collaboration (CKD-EPI) 2009 creatinine equation,[3] categorizing individuals as non-Black (consistent with previous Mexican population studies),[4] as it predicts GFR better than the 2021 CKD EPI equation.[5]

Mortality follow-up

Death registration in Mexico City is reliable and complete, with almost all deaths medically certified.[6] Participants are followed up for cause-specific mortality through probabilistic linkage based on name (including phonetic coding of names), age and sex to the Mexican System for Epidemiologic Death Statistics (*Subsistema Epidemiológico y Estadístico de Defunciones*

[SEED]), an electronic death registry in Mexico City administered by the Ministry of Health. Field validation of more than 7000 deaths matched in this way confirmed the reliability of the algorithm in more than 95% of cases. Diseases recorded on death certificates were coded using the International Statistical Classification of Diseases and Related Health Problems, Tenth Revision (ICD-10), with subsequent review by study clinicians to recode, when necessary, the underlying cause of death.[1] Participant deaths were tracked until 1st October 2022.

Covariates used in models

Where appropriate, the Cox proportional hazards regression models used throughout analyses were stratified by age-at-risk (5-year groups) and adjusted for sex (men or women), district of residence (Coyoacán or Iztapalapa), educational attainment (university or college, high school, elementary school, or other), smoking (never, former, less than daily, daily with <10 cigarettes per day, or daily with ≥10 cigarettes per day), leisure time physical activity (none, up to 2 times per week, or ≥3 times per week), weight, height, waist and hip circumference (each in 4 equally-sized groups according to the sex-specific distributions of each body measure), and diabetes (in five categories[7]: no diabetes, undiagnosed diabetes [no previously-diagnosed diabetes but HbA1c >6.5%], and previously-diagnosed diabetes [subdivided by HbA1c <9%, ≥9 to <11%, and ≥11%]).

Analyses were done in SAS version 9.4 and the figures were plotted in R version 4.2.2.

References

1. Alegre-Díaz, J. *et al.* Diabetes and Cause-Specific Mortality in Mexico City. *N. Engl. J. Med.* **375**, 1961–1971 (2016).
2. Soininen, P., Kangas, A. J., Würtz, P., Suna, T. & Ala-Korpela, M. Quantitative Serum Nuclear Magnetic Resonance Metabolomics in Cardiovascular Epidemiology and Genetics. *Circ. Cardiovasc. Genet.* **8**, 192–206 (2015).
3. Inker, L. A. *et al.* New Creatinine- and Cystatin C–Based Equations to Estimate GFR without Race. *N. Engl. J. Med.* **385**, 1737–1749 (2021).
4. Arreola-Guerra, J. M. *et al.* Performance of MDRD-IDMS and CKD-EPI equations in Mexican individuals with normal renal function. *Nefrol. Publicacion Of. Soc. Espanola Nefrol.* **34**, 591–598 (2014).
5. Gansevoort, R. T. *et al.* What should European nephrology do with the new CKD-EPI equation? *Nephrol. Dial. Transplant.* **38**, 1–6 (2023).
6. Mikkelsen, L. *et al.* A global assessment of civil registration and vital statistics systems: monitoring data quality and progress. *The Lancet* **386**, 1395–1406 (2015).
7. Herrington, W. G. *et al.* Effect of diabetes duration and glycaemic control on 14-year cause-specific mortality in Mexican adults: a blood-based prospective cohort study. *Lancet Diabetes Endocrinol.* **6**, 455–463 (2018).
